# An internal deletion of ADAR rescued by MAVS deficiency leads to a minute phenotype

**DOI:** 10.1093/nar/gkaa025

**Published:** 2020-01-20

**Authors:** Prajakta Bajad, Florian Ebner, Fabian Amman, Brigitta Szabó, Utkarsh Kapoor, Greeshma Manjali, Alwine Hildebrandt, Michael P Janisiw, Michael F Jantsch

**Affiliations:** 1 Department of Cell & Developmental Biology, Center for Anatomy and Cell Biology, Medical University of Vienna, Schwarzspanierstrasse 17, A-1090 Vienna, Austria; 2 Institute of Theoretical Biochemistry, University of Vienna, Währinger Strasse 17, A-1090 Vienna, Austria

## Abstract

The RNA-editing protein ADAR is essential for early development in the mouse. Genetic evidence suggests that A to I editing marks endogenous RNAs as ‘self’. Today, different *Adar* knockout alleles have been generated that show a common phenotype of apoptosis, liver disintegration, elevated immune response and lethality at E12.5. All the *Adar* knockout alleles can be rescued by a concomitant deletion of the innate immunity genes *Mavs* or *Ifih1* (MDA5), albeit to different extents. This suggests multiple functions of ADAR. We analyze *Adar^Δ7-9^* mice that show a unique growth defect phenotype when rescued by *Mavs*. We show that *Adar^Δ7-9^* can form a truncated, unstable, editing deficient protein that is mislocalized. Histological and hematologic analysis of these mice indicate multiple tissue- and hematopoietic defects. Gene expression profiling shows dysregulation of *Rps3a1* and *Rps3a3* in rescued *Adar^Δ7-9^*. Consistently, a distortion in 40S and 60S ribosome ratios is observed in liver cells. This dysregulation is also seen in *Adar*^Δ2-13^; *Mavs*^−/−^ but not in Adar*^E861A/E861A^*; *Ifih1^−/−^* mice, suggesting editing-independent functions of ADAR in regulating expression levels of *Rps3a1* and *Rps3a3*. In conclusion, our study demonstrates the importance of ADAR in post-natal development which cannot be compensated by ADARB1.

## INTRODUCTION

Adenosine to inosine deamination (A-to-I editing) in RNA is mediated by adenosine deaminases acting on RNA (ADARs) (1). ADARs bind dsRNAs and catalyze the deamination of adenosine to produce inosine (2,3). Formation of inosine in coding regions can change the genetic information of RNAs since inosine is primarily read as guanosine by the protein translation machinery (4,5). Apart from protein re-coding, editing affects splicing (6,7), stability of mRNAs, (8) and the biogenesis and processing of microRNAs (9–11). Moreover, massive editing is found in repeat-rich sequences that tend to form double-stranded structures with each other (12).

In mammals, two active ADARs are known today, termed ADAR1 (*Adar*) and ADAR2 (*Adarb1*) (13). In mammals, *Adarb1* (ADAR2) is mostly expressed in the nervous system and the gastrointestinal tract (14). In contrast, *Adar* (also known as *Adar1*) is ubiquitously expressed in two isoforms—an interferon inducible 150 kDa version (15) which is predominately cytoplasmic and a shorter constitutively expressed p110 kDa isoform which is nuclear (16). Both isoforms share common features like three dsRNA binding domains (dsRBDs), a nuclear localization signal (NLS) and a deaminase domain (17). However, ADAR1 p150, exclusively, has two Z-DNA binding domains and a nuclear export signal (NES) at its N-terminus while the shorter isoform has only one Z-DNA binding domain (ZBD) (18). The ZBDs and dsRBDs may confer additional functions that go beyond RNA-editing, such as RNA- and DNA-binding. Indeed, editing-independent functions of ADAR have been reported in microRNA biogenesis (10,19), neural induction (20), splicing (21) and translation (22).

Editing-independent functions of ADAR1 are also evident from the phenotypes of the different *Adar* alleles. Four different *Adar* knockout mice are reported in the literature depending on the number of exons deleted: Mouse *Adar* is composed of 15 exons and different number of exons are deleted in the different transgenic mouse lines. *Adar^Δ2-13^* deletes exons 2–13, *Adar^Δ7-9^* deletes exon 7–9, the isoform specific *p150^−/−^* allele selectively deletes exon 1 therefore only allowing for expression of the short p110 isoform (23–25). Finally, an enzymatically inactive allele *Adar^E861A/E861A^* has recently been created that expresses catalytically dead, but RNA-binding-competent ADAR (26). Interestingly, mice with a deletion in *Adar* die around day 12.5 *in utero* (23–25), except the catalytically-dead point mutation allele *Adar^E861A/E861A^* displays embryonic lethality at E13.5 (26). The phenotypes of all embryonically lethal *Adar* alleles is comparable and is accompanied by liver disintegration, increased apoptosis and a massive upregulation of interferon stimulated genes (ISGs) (23,24,26). Seemingly, lack of editing activity is solely responsible for the observed immune response: the mouse carrying the catalytic-dead point mutation *Adar^E861A/E861A^* shows the same immune response as a full *Adar* deletion. Therefore, the contribution of other domains in the ADAR1 protein to the immunological phenotype seems marginal. The observed immune signaling is centered on the MDA5/MAVS pathway, as a concurrent deletion of *Ifih1* in *Adar^E861A/E861A^* completely rescues lethality. *Adar^E861A/E861A^; Ifih1^−/−^* are fertile, live till adulthood and have no reported defects in hematopoiesis, apoptosis, or in other tissues (27).

Interestingly, the elevated immune response of *Adar^Δ2-13^* and *Adar p150^−/−^* mice is also rescued by deletion of *Mavs* or *Ifih1* (28,29). Still, lethality of these knockout mice is only partially rescued and both of them show a unique phenotype. *Adar*^Δ2-13^*; Mavs*^−/−^ pups die within 24 h of birth and show elevated apoptosis (28,29) while *Adar p150^−/−^; Mavs^−/−^* live up to weaning and show defects in hematopoiesis (29). Together, these reports indicate that editing independent functions of *Adar* regulate apoptosis and hematopoiesis. However, it is still unclear if elevated apoptosis and defects in hematopoiesis are reasons for the early lethality of *Adar^Δ2–13^*; *Mavs^−/−^* and whether other cellular functions are also affected.

To gain closer insight on additional phenotypes, we analyze here an Adar allele that deletes exons 7–9 (23). The *Adar^Δ7-9^* allele was considered identical to the complete deletion of *Adar (Adar^Δ2–13^*) (23). Interestingly, we show that rescue of the *Adar^Δ7-9^* allele by *Mavs^−/−^* or I*fih^−/−^* gives rise to a phenotype that is intermediate of *Adar^Δ2-13^; Mavs^−/−^* and *Adar^E861A/E861A^; Ifih1^−/−^*. We show here that *Adar^Δ7-9^* can form a truncated, unstable and mislocalized ADAR1 protein. *Adar^Δ7-9^; Mavs^−/−^* mice show various tissue-specific defects. However, a common feature found in all tissues studied is de-regulation of the 40S ribosomal protein RPS3a1, and its pseudogene RPS3a3. Consistent with this, the rescued mice showed accumulation of free 60s ribosomal subunits in sucrose gradient profiling of ribosomes. *Rps3a1* and *Rps3a3* are also de-regulated in *Adar^Δ2-13^; Mavs^−/−^* but not in the fully rescued *Adar^E861A/E861A^; Ifih1^−/−^* suggesting that ADAR1 regulates *Rps3a1* and *Rps3a3* independent of editing.

## MATERIALS AND METHODS

### Mouse breeding

*Adar^Δ7-9^* and *Adarb1^−/−^; Gria2^R/R^* were kindly provided by Dr. Peter Seeburg (23,30). Both these genotypes were kept in a 129/Sv background. *Mavs^−/−^* (B6;129-Mavs^tm1Zjc^/J; Stock No.: 008634) (31) and *IFIH1^−/−^* (B6.Cg-Ifih1^tm1.1Cln^/J; Stock No.: 015812) (32) were purchased from Jackson laboratories. All experiments were done in accordance with the animal ethics guidelines of Medical University of Vienna following FELASA, national, and European animal welfare laws.

### Histology

Spleens, kidneys, intestines and hearts were isolated from littermates 15 days post-partum, fixed overnight in 4% paraformaldehyde, dehydrated, embedded in paraffin and 4 μm sections were taken. Hematoxylin and eosin (H&E) staining was carried out following standard protocols. Microscopic analysis and imaging were performed using an Olympus BX61VS slide scanner and OlyVIA 2.9 (Olympus) software.

### Flow cytometry

Red blood cells of bone marrow and spleen were lysed using hypotonic shock and washed twice with PBS. To exclude dead cells, samples were stained with 7-AAD Viability Staining Solution (eBioscience, San Diego, CA, USA), prior to Fc blocking with TruStain FcX™ anti-mouse CD16/32 (BioLegend, San Diego, CA, USA). Suspensions were stained for cell surface proteins with appropriate combinations of the following monoclonal antibodies conjugated to allophycocyanin, redFluor™ 710, allophycocyanin-eFluor 780 conjugate, brilliant violet 421, brilliant violet 605, fluorescein isothiocyanate, peridinin chlorophyll protein-cyanine 5.5, phycoerythrin and phycoerythtrin-cyanine7: anti-Ly6G (1A8, BioLegend), anti-Ly6C (HK1.4, BioLegend), anti-CD3 (17A2, Tonbo Biosciences, San Diego, California), anti-CD8a (53-6.7, Tonbo Biosciences), anti-B220 (RA3-6B2, Tonbo Biosciences), anti-CD19 (6D5, BioLegend), anti-NK1.1 (PK136, ebioscience), anti-CD4 (RM4-5, ebioscience, San Diego, CA, USA), anti-F4/80 (BM8, BioLegend), anti-MHCII (M5/114.15.2, Tonbo Bioscience), anti-CD11c (N418, ebioscience) and anti-CD11b (M1/70, ebioscience). AnnexinV Apoptosis Detection Kit PE (eBioscience) was used according to the manufacturer's protocol. Dead cells were excluded during analysis based on their light-scattering characteristics and 7-AAD staining. Cell doublets were excluded based on FSC-H/FSC-A and SSC-H/SSC-A. All data acquisitions were performed using a CytoFLEX S flow cytometer (Beckman Coulter, Fullerton, CA, USA) interfaced with CytExpert 2.0. FlowJo X (Tree Star, Ashland, OR, USA) software was used for data analysis and graphical representation.

Exploratory data analysis, visualization and statistical testing was performed with Prism 5 (GraphPad Software) unpaired two-tailed Student's *t* test. Means are depicted as horizontal bars, respectively. Statistical significance is indicated as follows: *P < 0.05; **P < 0.01; ***P < 0.001.

### Western blotting and antibodies

For western blotting HeK293 cells or MEFs were grown in DMEM/FBS/Pen-Strep. To induce the production of ADAR1, cells were transfected with FLAG-tagged ADAR1p150 fused to T2A-GFP in pcDNA 3.1.

For induction of endogenous ADAR1, cells were treated over night with mouse IFN-**α** (Hycult Biotech, Uden, Netherlands) to 1000 units/ml, final concentration. After over-night expression, cells were washed and directly lysed in 2× SDS sample buffer (33). Cell lysates were sonicated to shear DNA, denatured at 98°C and loaded onto a 7% SDS protein gel. After blotting onto nitrocellulose membrane, proteins were detected with an anti FLAG-antibody, or with mouse mAb 15.8.6 (1:300, Santa Cruz Biotechnology, order nr. sc-73408) directed against ADAR1. Blots were further developed with secondary HRP-coupled antibodies (1:5000, Pierce, 31444) and detected via chemiluminescence (WesternBright Sirius, advansta, Menlo Park, CA, USA). Alternatively, blots were detected with a rabbit anti ADAR1-p150 antibody (1:1000, SynapticSystems, order nr. 293003) and goat anti-Rabbit IgG1 HRP conjugate (1:5000, Cell Signaling Technologies, order nr. 7074).

### qRT-PCR

RNA was isolated using TriFast™ (Peqlab, Erlangen, Germany) according to manufacturer's instructions. Followed by treatment with DNaseI (New England Biolabs, Ipswich, Massachusetts), RNA was purified by Phenol:Chloroform extraction. 500ng of RNA was reverse transcribed using M-MuLV Reverse Transcriptase (New England Biolabs, Ipswich, MA, USA). qRT-PCR was performed using GoTaq® qPCR Master Mix (Promega, Wisconsin, United States) on Biorad a CFX Connect™ Real-Time PCR Detection System (BioRad, Hercules, CA, USA).

### RNA-Seq library preparation

Liver was harvested from P15 old mice and homogenized in TriFast™ (Peqlab, Erlangen, Germany) by cutting into small pieces and passing through a syringe with a 18-G needle. Bone marrow cells were isolated by flushing the femur and tibia of the P15 mice with PBS. RNA was isolated following the manufacturer's instructions for TriFast™, treated with DNaseI (New England Biolabs, Ipswich, MA, USA) and then purified by Phenol:Chloroform extraction. Ribosomal RNAs were removed using the Ribo-Zero rRNA removal kit (Illumina, San Diego, CA, USA) and cDNA libraries were subsequently generated using the NEBNext^®^ Ultra™ Directional RNA Library Prep Kit for Illumina^®^ (New England Biolabs, Ipswich, MA, USA) and sequenced in a paired-end mode with 125 bp read length on a HiSeq2500 (Illumina, San Diego, CA, USA) machine at a read depth of 31 million reads per sample.

### Analysis of RNA-Seq data

Sequence reads were quality trimmed and adaptor clipped using trimmomatic (v0.33) (34). Quality was monitored before and after using FastQC. For quantification of transcript abundance (TPM), a reference transcriptome was obtained from ENSEMBL Biomart for mus musculus (GRCm38.p4) (35) and used together with salmon (v0.9.1) (36) applying sequence-specific and fragment GC bias correction. Resulting read count estimates were used with bioconductor packages tximport (37) and DESeq2 (38) to call differentially expressed genes. Coverage plots for *Rps3a1* and *Rps3a3* were produced by mapping the trimmed reads against a reference transcriptome containing the mature mRNAs of *Rps3a1* and *Rps3a3* including 1 kb of flanking regions (since no UTR annotation is available for *Rps3a3*) using the tool segemehl (v0.2) (39) requiring an mapping accuracy of 0.95. Coverage across the gene body was deduced with tool genomeCoverageBed (40).

### Availability of RNA-Seq data

The GEO Accession number for RNA- sequencing data generated for this project and the publicly available data used in this project, are given below:

**Table:** 

*Adar^Δ2-13^* RNA-Seq (28)	GSE62917
*Adar^E861A/E861A^; Ifih1^−/−^* RNA-Seq (27)	GSE94387
Early craniofacial RNA-Seq (41)	GSE55966
This manuscript (liver and bone marrow RNA-Seq)	PRJEB31568
This manuscript (cortex RNA-Seq)	PRJEB31565

Moreover, ENCODE RNA-Seq data (polyA and total RNA) from wildtype mice at 10 weeks of age and at P0 stage was also analyzed.

### Polysome profiling

Liver harvested from P15 old mice was homogenized in polysome lysis buffer (15 mM Tris–HCl pH 7.4, 15 mM MgCl_2_, 300 mM NaCl, 1% Triton X-100, 0.1% ß-mercaptoethanol, RNase Inhibitor [New England Biolabs, Ipswich, MA, USA], cOmplete™ mini protease inhibitor cocktail [Roche, Basel, Switzerland] and cycloheximide 100 ug/ml). Mock transfected and Flag-RPS3a3 transfected HeK293T were treated with cycloheximide (100 ug/ml) for 10 min at 37°C. Cells were washed with ice-cold PBS with 100 ug/ml cycloheximide and lysed in polysome lysis buffer by passing 3-times through a 26-G needle. The lysates were cleared by centrifugation at 20 000g for 30 min. The lysate was layered on top of 10–50% sucrose gradients and centrifuged in a SW40Ti ultracentrifuge rotor at 35 000 rpm for 2 h. Gradients were factionated and OD_254_ measurements were taken. For HeK293T with over-expressed Flag-RPS3a3, 500 μl fractions were concentrated to 100 μl in Amicon^®^ 10K centrifugal filter devices (Millipore™). 25 μl concentrated material was loaded on 12% polyacrylamide gels for western blotting.

## RESULTS

### Deletion of *Mavs* or *Ifih1* (MDA5) rescues *Adar^Δ7-9^* mice to the same extent

IRF3 is a transcription factor which induces the production of IFN-beta to amplify the IFN response, upon viral infection (42). IRF3 is also involved in amplifying immune signaling upon transfection of unedited dsRNAs (43). This prompted us to study its contribution in immune signaling of *Adar^Δ7-9^* mice. To do so, we bred heterozygous *Adar^+/Δ7-9^; Irf3^−/−^* mice. No live, homozygous *Adar ^Δ7-9/Δ7-9^; Irf3^−/−^* progeny was obtained from these crosses (Supplementary Figure S1A). Embryo collection at various stages of gestation revealed that *Irf3* deletion rescued *Adar^Δ7-9^* mice by only one day. *Adar^Δ7-9^; Irf3^−/−^* embryos at E13.5 could be obtained but showed strong growth retardation (Supplementary Figure S1B). Consistently, mendelian ratios of the offspring became more distorted with advanced development (Supplementary Figure S1A). At E11.5, E13.5 and E 14.5 embryos presented an expected Mendelian ratio while no *Adar* deficient life pups were obtained (Supplementary Figure S1A). Interestingly, deletion of *Irf3* did not alleviate the immune response seen in *Adar^Δ7-9^* mice. Already at E11.5, *Adar^Δ7-9^; Irf3^−/−^* and *Adar^Δ7-9^* embryos showed comparable expression of Interferon-Stimulated Genes (ISGs) (Supplementary Figure S1C).

*Adar^Δ2–13^* can be rescued until birth by a concomitant deletion of MAVS (28). The point mutation *Adar^E861A/E861A^; Ifih1^−/−^*^,^ in contrast, is fully viable (26). To understand to which extent *Mavs^−/−^* and *Ifih1^−/−^* can rescue *Adar^Δ7-9^* mice, we crossed *Mavs^−/−^* and *Ifih1^−/−^* with *Adar^+/Δ7-9^* to generate *Adar*^Δ7-9^*; Mavs*^−/−^ and *Adar*^Δ7-9^*; Ifih1*^−−/−^ mice. The generation and phenotype of the *Adar^Δ7-9^* mice has been previously described (23). In short, *Adar^Δ7-9^* mice are editing deficient and die at E12.5 showing heightened immune response. At E12.5, the phenotype of *Adar^Δ2–13^* and *Adar^Δ7-9^* is indistinguishable (23). In the crosses tested, *Mavs^−/−^* or *Ifih1^−/−^* deletions rescued the embryonic lethality of *Adar^Δ7-9^* mice. However, in our hands the phenotype of both *Adar^Δ7-9^; Mavs^−/−^* and *Adar^Δ7-9^, Ifih1^−/−^* was different than that of the previously reported rescue experiments on this and other Adar alleles.

**Figure 1.**
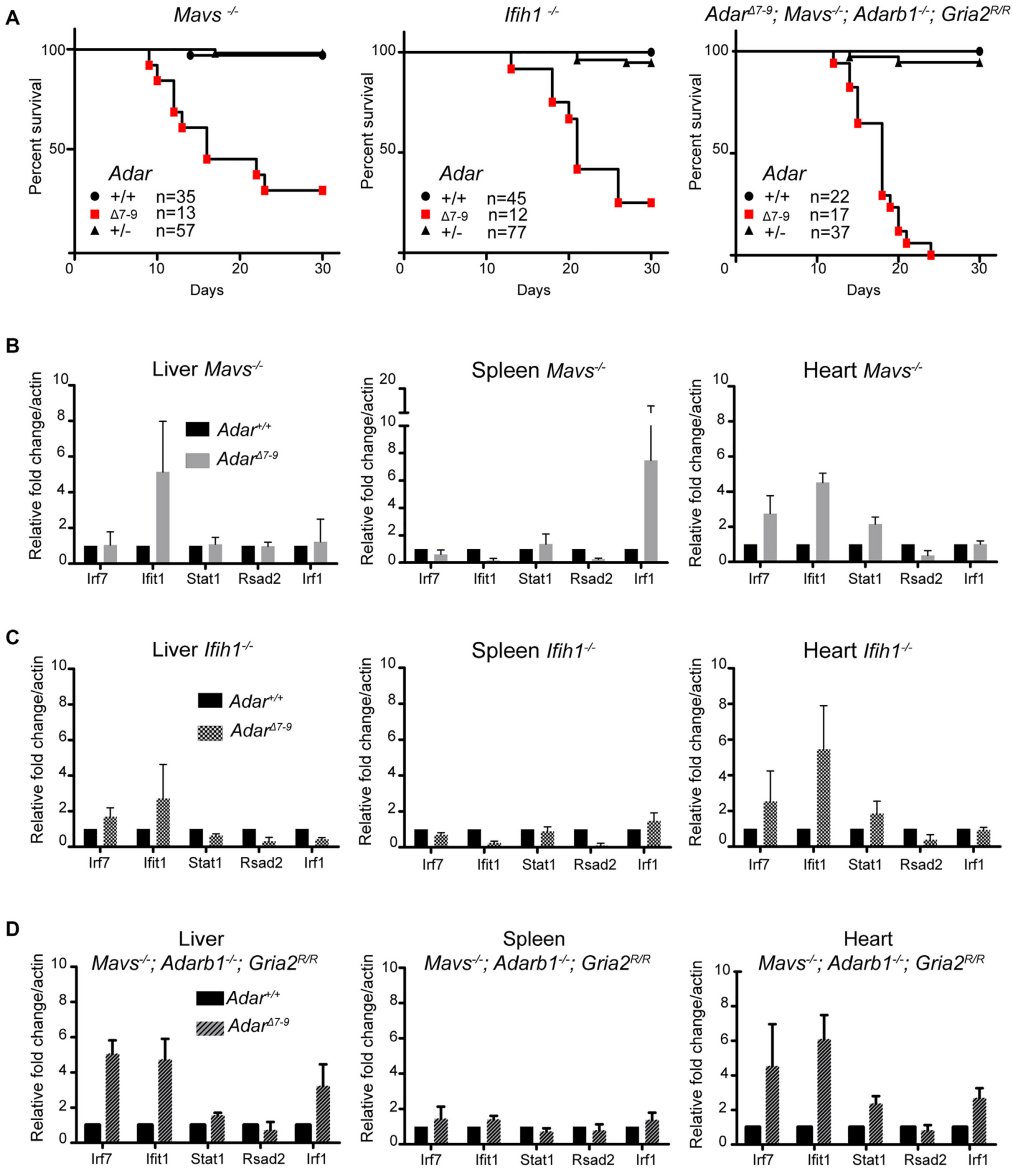
Rescue of *Adar^Δ7-9^ by Mavs^−/−^*, *Ifih1^−/−^* and *Mavs^−/−^; Adarb1^−/−^; Gria2^R/R^*. (**A)** Post-natal survival curves of *Adar^Δ7-9^; Mavs^−/−^*, *Adar1^Δ7-9^; Ifih1^−/−^* and *Adar^Δ7-9^; Mavs^−/−^; Adarb1^−/−^; Gria2^R/R^* mice. Expression levels of 5 Interferon stimulated genes measured by qPCR in three different tissues of (**B**) *Adar^Δ7-9^; Mavs^−/−^* (**C**) *Adar^Δ7-9^; Ifih1^−/−^* and (**D**) *Adar^Δ7-9^; Mavs^−/−^; Adarb1^−/−^; Gria2^R/R^* mice.

The rescued mice showed phenotypes of apparently different penetrance. Analysis of sequencing data from three different tissues from these mice indicated that they were indeed devoid of ADAR1 activity (Supplementary Table S1). While most mice lived until ∼15 days after birth, a few mice survived for several months (the longest living mouse stayed alive until >18 months after birth and was then sacrificed for histological analysis-see below). *Adar^Δ7-9^; Mavs^−/−^* and *Adar^Δ7-9^; Ifih1^−/−^* pups are phenotypically similar, both are developmentally retarded, significantly smaller than their wild-type counterparts and generally immobile (Supplementary Figure S2A). Survival curves revealed that 69.24% of *Adar^Δ7-9^; Mavs^−/−^* and 75% of *Adar^Δ7-9^; Ifih1^−/−^* die by day 30 after birth (Figure 1A). The remaining pups survive beyond 30 days after birth. Out of these, two *Adar^Δ7-9^; Mavs^−/−^* lived up to six months and one survived at least till 18 months, while no *Adar^Δ7-9^; Ifih1^−/−^* lived till 6 months of birth. *Adar^Δ7-9^; Mavs^−/−^* and *Adar^Δ7-9^; Ifih1^−/−^* showed deviation from the expected Mendelian ratios (Supplementary Figure S2B). However, it is unclear if the distortion in ratio is due to incomplete rescue of the phenotype resulting in embryonic lethality, or if the rescued mice were cannibalized immediately after birth.

To test whether the partially rescued mice showed any expression of interferon stimulated genes (ISGs) we tested ISG expression in various tissues of *Adar^Δ7-9^; Mavs^−/−^* and *Adar^Δ7-9^; Ifih1^−/−^* mice. Heart, spleen and liver tissues from these rescued mice showed a mild immune response compared to the respective *Mavs^−/−^* and *Ifih1^−/−^* littermates (Figure 1B and C). Consistent with their comparable survival curves, ISG expression was similar in both *Adar^Δ7-9^; Mavs^−/−^* and *Adar^Δ7-9^; Ifih1^−/−^* (Figure 1B and C). This demonstrated that *Mavs^−/−^* and *Ifih1^−/−^* not only rescues the lethality of *Adar^Δ7-9^* mice to the same extent but also alleviates the immune response associated with *Adar^Δ7-9^* to a similar level. In summary, we show that the phenotype of Adar^Δ7-9^*; Mavs^−/−^* mice can be less severe than previously reported and is less severe than that of *Adar^Δ2-13^; Mavs^−/−^* which reportedly die immediately after birth but is more severe than *Adar^E861A/E861A^*; *Ifih1^−/−^* that are fully rescued (26,28,29).

### *ADARB1* minimally rescues *ADAR* deficiency

Since, ADAR and ADARB1 proteins have a highly conserved deaminase domain and also have overlapping targets (44,45), we wondered whether ADARB1 can compensate for the absence of ADAR. For this, we used *Adarb1^−/−^* mice rescued by a point mutation in Gria2 (*Adarb1^−/−^; Gria2^R/R^*) (30). In these mice, the genomic copy of Gria2 mimics the edited state by carrying a guanosine instead of the edited A, thereby replacing a genomically encoded glutamine (Q) by an edited arginine (R) in the encoded protein. *Adarb1^−/−^; Gria2^R/R^* mice live and breed normally but show significant differences in gene expression, physiology and behavior (30,46). We crossed *Adarb1^−/−^; Gria2^R/R^* mice with *Adar^+/Δ7-9^; Mavs^−/−^* to obtain *Adar^Δ7-9^; Mavs^−/−^*; *Adarb1^−/−^*; *Gria2^R/R^* (*Adar; Adarb1* rescued). *Adar; Adarb1* rescued mice were born at expected Mendelian frequencies (Supplementary Figure S2B). Upon macroscopic investigation, these mice were minute and indistinguishable from *Adar^Δ7-9^; Mavs^−/−^* and *Adar^Δ7-9^; Ifih1^−/−^* (Supplementary Figure S2A). 35.3% of *Adar; Adarb1* rescued mice died by 15 days after birth. However, in contrast to *Adar^Δ7-9^; Mavs^−/−^*, none of the *Adar^Δ7-9^; Mavs^−/−^; Adarb1^−/−^; Gria2^R/R^* mice survived beyond 30 days of birth (Figure 1A). Expression analysis of ISGs in heart, spleen and liver of *Adar^Δ7-9^; Mavs^−/−^; Adarb^−/−^; Gria2^R/R^* showed mild immune response at levels similar to *Adar^Δ7-9^; Mavs^−/−^* tissues (Figure 1B–D). Thus, ADAR1B shows a minor compensation for the absence of ADAR1 in survival and also in alleviating the immune response.

### *Adar^Δ7-9^* forms a truncated, editing-deficient, mislocalized protein

**Figure 2.**
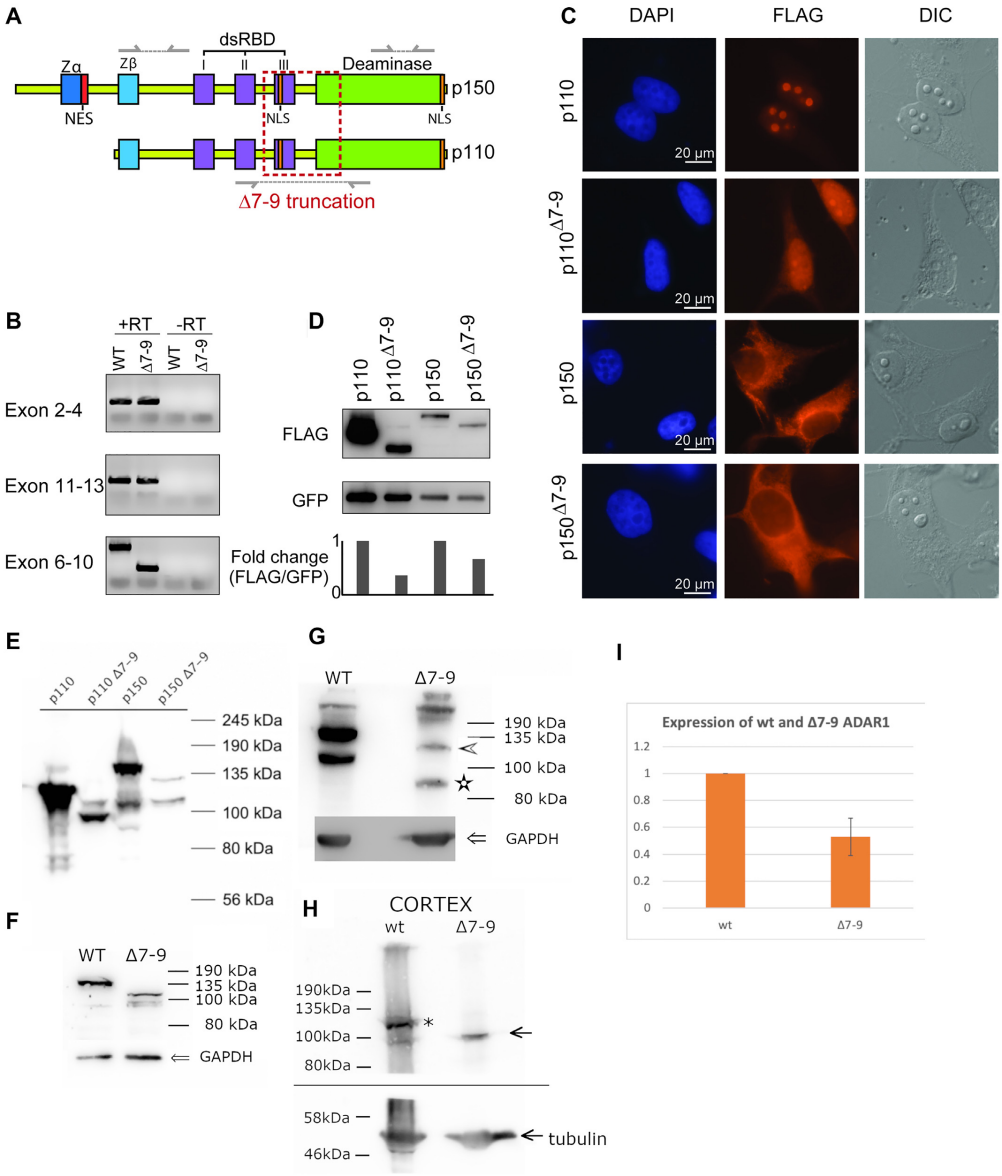
ADAR1 variants without exons 7–9 can be expressed in cells. (**A**) Illustration of the p150 and p110 isoforms of ADAR1 indicating the deleted parts of the *Adar^Δ7-9^* allele (red box). The truncation affects only the third double-stranded RNA binding domain (dsRBD), the embedded nuclear localization signal (NLS) and the deaminase domain. Z-DNA binding domains (Zα and Zβ), the nuclear export signal (NES), dsRBD 1 and 2 as well as a C-terminal NLS motif are unaffected. Amplicons tested for detecting truncation are indicated. (**B**) RT-PCR analysis of wildtype and *Adar^Δ7-9^* transcript in primary MEFs isolated at E11.5. (**C**) Fluorescence microscopy images of N-terminally FLAG-tagged and C-terminally fused with 2A-eGFP versions of ADAR1 (p110, p110^Δ7-9^, p150 and p150^Δ7-9^) transfected into HeLa cells. DAPI shows nuclear DNA and FLAG-tagged fusions are visualized in the mCherry channel. (**D**) Western blot analysis of whole-cell extracts of HeLa cells transfected as in (C) using FLAG antibody to visualize fusion proteins (upper panel). eGFP was used for normalization (lower panel). (**E**) Detection of transfected constructs used in (C) with monoclonal anti-ADAR1 antibody 15.8.6 by western blotting. (**F**) Detection of ADAR1 in wt and ADAR1^Δ7-9^ -derived MEFs after IFN induction using a polyclonal antibody directed ADAR1 p150. (**G**) Detection of ADAR1 in wt and ADAR1^Δ7-9^ -derived MEFs after IFN induction using a monoclonal pan-anti ADAR1 antibody. Arrowhead marks size of predicted p150^Δ7-9^ protein and asterisk depicts the size of the p110^Δ7-9^ protein. (**H**) Detection of ADAR1 in wt and ADAR1^Δ7-9^ brain lysates. tubulin marks detection of the lower part of the blot with an anti-tubulin antibody. **(I)** Quantification of ADAR p110 in three independent blots of cell lysates indicates ∼20% expression of p110^Δ7-9^ protein

Since the phenotype of both *Adar^Δ7-9^; Mavs^−/−^* and *Adar^Δ7-9^; Ifih1^−/−^* was less severe than that of *Adar^Δ2-13^; Mavs^−/−^* but more severe than the fully rescued *Adar^E861A/E861A^; Ifih1^−/−^* we wondered whether *Adar^Δ7-9^* could form a truncated protein. *In-silico* analysis predicted that deletion of *Adar* exons 7–9 is in-frame (Supplementary Figure S3). Deletion of exons 7–9 disrupts part of the deaminase domain and the third RNA binding domain (dsRBD3). dsRBD3 embeds a nuclear localization signal (NLS) in human ADAR1 while mouse ADAR1 also harbors a C-terminal NLS (47,48,50). The other two RNA binding domains and the ZBDs remain intact in the *Adar^Δ7-9^* allele (Figure 2A). Indeed, using primers spanning exons 6 and 10 of *Adar* cDNA, we were able to detect a truncated *Adar^Δ7-9^* transcript in primary MEFs (Figure 2B). Sanger sequencing verified the truncation to be in-frame. To determine whether the ADAR^Δ7-9^ could produce a protein we cloned the ADAR1 isoforms p110 and p150 and their truncated Δ7-9 counterparts (accession no. AAK16102.1). The cloned cDNAs were N-terminally FLAG-tagged and C-terminally fused with a self-cleaving 2A peptide to eGFP (49). Overexpression of these variants confirmed the nuclear localization of ADAR1 p110 and cytoplasmic localization of ADAR1 p150. p110^Δ7-9^ still accumulated in the nucleus but showed a less prominent nucleolar localization, suggesting that human and mouse ADAR1 proteins may differ with respect to their nuclear localization signals. In fact, it was shown previously that deletion of a region surrounding the third dsRBD interferes with nucleolar association of mouse ADAR1 while a C-terminal motif was mapped to be crucial for nuclear localization of mouse ADAR1 (50). The localization of p150^Δ7-9^ resembled the cytoplasmic localization of full-length p150 (Figure 2C). Western blot analysis revealed that all four protein versions migrated with the expected patterns, confirming that p110^Δ7-9^ and p150^Δ7-9^ are expressed when transiently transfected, with no detectable degradation products (Figure 2D). Interestingly, normalization to the co-translated eGFP indicated that the truncated variants are less stable *in cellulo*, especially p110^Δ7-9^ is more unstable compared to its full-length counterpart p110 (Figure 2D).

Next, we tested whether full length ADAR1 and ADAR1^Δ7-9^ could be detected in MEFs or organs of wt or *Adar*^Δ7-9^ mice. We therefore tested commercial monoclonal anti *Adar* antibody 15.8.6 (Santa Cruz) on HeK293 cells that were transfected with the above mentioned FLAG-tagged ADAR1 p110, p150, p110^Δ7-9^ and p150^Δ7-9^ constructs. Indeed, the 15.8.6 antibody could detect all four recombinantly expressed versions of the protein (Figure 2E).

Subsequently, we tested whether full length and truncated p150 and p110 versions of ADAR1 could be detected in two different isolates of MEFs derived from wild-type and mutant *Adar*^Δ7-9^ mice. This was done using an antibody against the p150 version of ADAR1 (Synaptic Systems) (Figure 2F) or by using a pan-ADAR1 antibody (15.8.6 / Santa Cruz) (Figure 2G). To induce expression of ADAR1, cells were treated over night with IFN**α**. Indeed, all four versions of the protein could be detected expressed from the endogenous locus (Figures 2F, G).

To test, whether the ADAR1^Δ7-9^ could also be detected in mice, we tested several organs for the expression of full-length and ADAR1^Δ7-9^. To this end, ADAR1^Δ7-9^ could be most convincingly detected in brains (Figure 2H, Supplementary Figure S4). Thus, ADAR1^Δ7-9^ can give rise to a truncated protein. It should be noted, however, that expression of the ADAR1^Δ7-9^ derived proteins was much weaker (about 40%) than that of full-length protein, both in cells and organs (Figure 2I).

### *Adar^Δ7-9^; Mavs^−/−^* show defects in multiple organs

**Figure 3.**
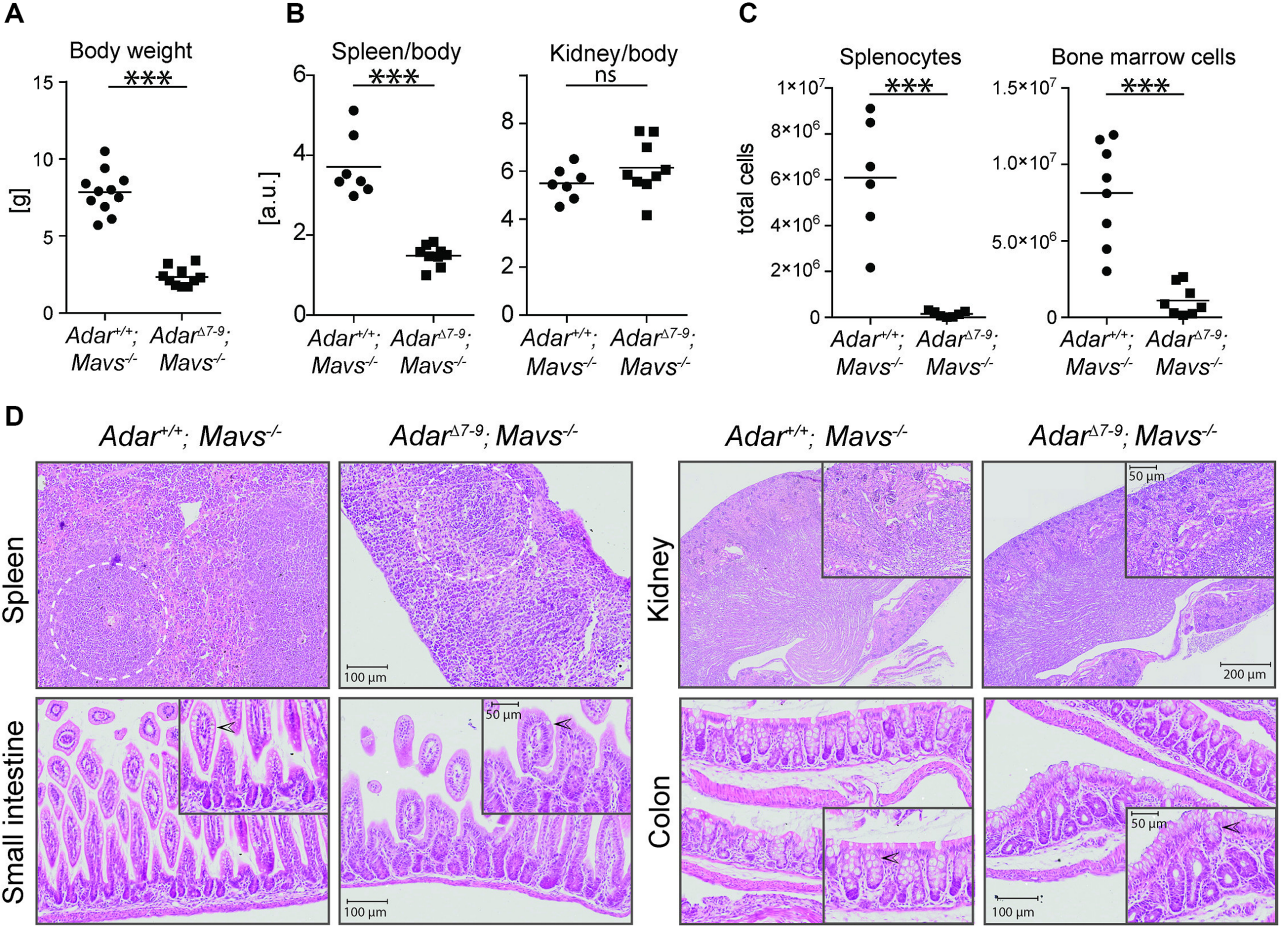
Phenotypic analysis of *Adar^Δ7-9^; Mavs^−/−^* mice at P15. (**A**) Dot plot showing reduced body weight of *Adar^Δ7-9^; Mavs^−/−^* compared to *Adar^+/+^; Mavs^−/−^* mice (*n* = 11 per genotype). (**B**) Normalization of spleen (left) and kidney (right) weight to total body weight. A specific effect is seen on spleen, but not the kidney of *Adar^Δ7-9^; Mavs^−/−^* mice (n = 7 *Adar^+/+^; Mavs^−/−^* and n = 9 *Adar^Δ7-9^; Mavs^−/−^*). (**C**) Dot plots showing reduced splenocytes (left, *n* = 6/genotype) and bone marrow cells (right, *n* = 8/genotype) in *Adar^Δ7-9^; Mavs^−/−^* compared to *Adar^+/+^; Mavs^−/−^* mice. Horizontal bars in (A) – (C) represent the mean; ***P < 0.001; ns = non-significant as determined by unpaired Student's *t* test. (**D**) Three mice per genotype were analyzed histologically at day 15 post-partum. Analyzed organs were spleen, small intestine, colon and kidney. Note the diminished cellularity of the spleen and the underdeveloped germinal center (dotted circle) in *Adar^Δ7-9^; Mavs^−/−^* mice. In the small intestine and to a lower extent in the colon, goblet cell (denoted by arrowhead) number is reduced in in *Adar^Δ7-9^; Mavs^−/−^* mice. Shown are representative sections in 8× magnification (20× in inlays) of spleen, small intestine and colon and 2× magnification (8× in inlays) of kidney with scale bars indicated.

Since *Adar^Δ7-9^; Mavs^−/−^* and *Adar^Δ7-9^; Ifih1^−/−^* did not show any phenotypic differences and *Adar^Δ7-9^; Irf3^−/−^* could not survive beyond embryonic day E13.5, we focused on *Adar^Δ7-9^; Mavs^−/−^* mice for further analysis. *Adar^Δ7-9^; Mavs^−/−^* mice were about 3.5-fold smaller than their control littermates (Figure 3A). Accordingly, kidney and spleen of these animals displayed a reduction in weight. To understand whether these differences were simply due to reduced overall body size or whether an organ is specifically affected, organ weights were normalized to body weights of the respective animals. This revealed, that the spleen is specifically affected in these animals, whereas the kidney weight is in a comparable ratio to control animals (Figure 3B). *Adar^Δ7-9^; Mav^−/−^* mice showed a strong reduction in the cellularity of the spleen and the bone marrow by 23- and 5-fold, respectively (Figure 3C). The different isoforms of ADAR1 were shown to control development of organs such as kidney, spleen, lymph nodes and intestine, independent of inhibition of interferon-production (29). Histological examination of selected organs at day 14 post-partum revealed pleiotropic effects. In line with a previous study, cellularity of the spleen was generally low (Figure 3D). Similarly, intestinal development and homeostasis were also disturbed throughout the intestine, with strongest effects observed in the small intestine (29). Goblet cells in *Adar^Δ7-9^; Mavs^−/−^* mice were preferentially located at the luminal part of the epithelium, whereas they were distributed along the whole villi and crypts of the small intestine and the colon in wild type mice, respectively. Additionally, mild signs of villar fission/fusion and inflammation were detected (Figure 3D). We could not detect a profound change in the organization of the kidneys in these animals (Figure 3D). The residual inflammatory signature in the heart of *Adar^Δ7-9^; Mavs^−/−^* mice (Figure 1B) is also not reflected on the histological level, as this tissue appeared comparable to the *Adar^+/+^; Mavs^−/−^* littermates (Supplementary Figure S5). Interestingly, the penetrance of the histological phenotype seems variable. We have also performed histological examination of the kidneys and spleen of an 18 month-old *Adar^Δ7-9^; Mavs^−/−^* long-time survivor and its heterozygous *Adar^Δ7-9/+^; Mavs^−/−^* sibling. However, in this long-time survivor the spleen and liver appeared morphologically normal, indicating that the penetrance of the *Adar^Δ7-9^; Mavs^−/−^* phenotype is variable (Supplementary Figure S6).

**Figure 4.**
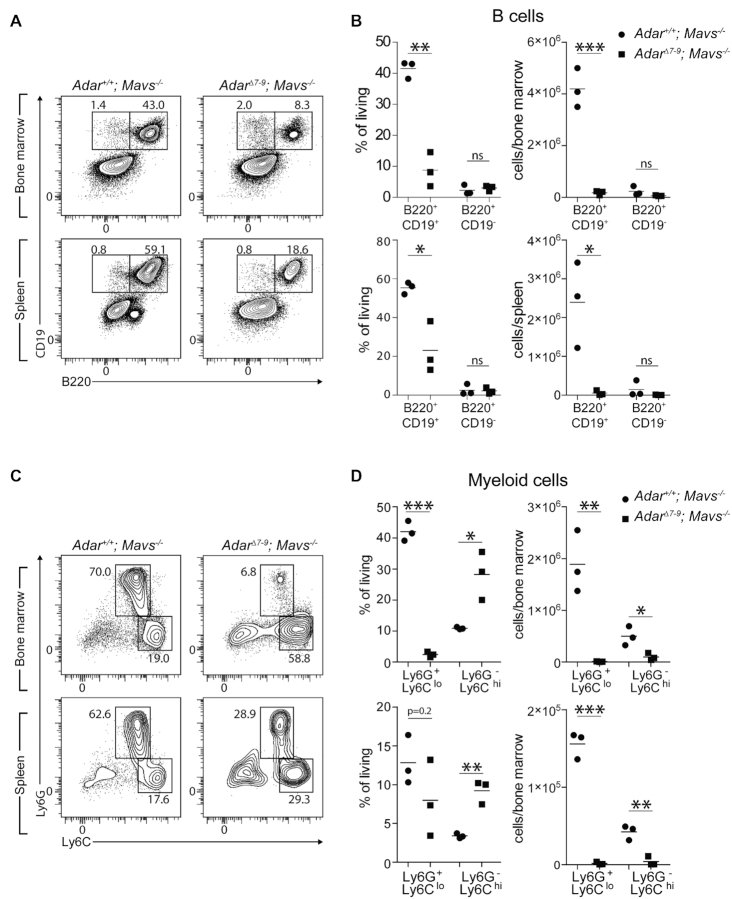
Altered B cell and neutrophil numbers in P15 Adar^Δ7-9^; *Mavs^−/−^* mice. (**A** and **B**) Immature and mature B cells in bone marrow (upper panels) and spleen (lower panels) were analyzed by flow cytometry. (A) Representative flow plots of bone marrow (upper panel) and spleen (lower panel) from Adar1^+/+^; Mavs^−/−^ (left panels) and Adar^Δ7-9^; Mavs^−/−^ (right panels) animals, subgated for living NK1.1^−^ cells. Numbers next to the outlined areas indicate percentages within this population. (B) Dot plots showing mature (B220^+^CD19^+^) and immature (B220^+^CD19^−^) B cells in the bone marrow (upper panel) and spleen (lower panel). Percentages of living cells (left panels) were used to determine total cell numbers (right panels). (**C** and **D**) Neutrophils and inflammatory monocytes were analyzed in bone marrow and spleen of Adar1^+/+^; Mavs^−/−^ and Adar^Δ7-9^; Mavs^−/−^ animals. (C) Representative flow plots of subgated for living, CD11b^+^ cells. Numbers next to the outlined areas indicate percentages within the CD11b^+^ population. (D) Dot plots showing neutrophils (Ly6G^+^Ly6C^lo^) and inflammatory monocytes (Ly6G^−^ Ly6C^hi^). Percentages of living cells (left panel) were used to determine total cell numbers (right panel). *n* = 3 mice/genotype; horizontal bars in (C) and (D) represent the mean; *P < 0.05, **P < 0.01 and ***P < 0.001 determined by unpaired Student's *t* test.

A previous study showed that *Adar^−/−^; Mavs^−/−^* animals have specifically reduced splenic B cell numbers controlled by the p150 isoform (29). This study and the reduced cellularity of the bone marrow and the spleen (Figure 3C) prompted us to analyze hematopoietic organs in more detail. Flow cytometric analysis of bone marrow and spleen revealed a specific loss of the mature B cell population (B220^+^CD19^+^) in *Adar^Δ7-9^; Mavs^−/−^* animals compared to their control littermates (Figure 4A). Mature B cell numbers were reduced 24- and 50-fold in the bone marrow and the spleen, respectively (Figure 4B). Intriguingly, the number of immature B cells (B220^+^CD19^−^) was not changed (Figure 4B), indicating a specific loss of mature cells either due to e.g. apoptosis or problems of proper homing. The bone marrow is also the primary site of differentiation of circulating myeloid cells, like inflammatory monocytes and neutrophils (51). Contributions of these two cell types to the reduction of bone marrow cellularity were determined by flow cytometry. The fraction of neutrophils (CD11b^+^Ly6G^+^Ly6C^lo^) was strongly diminished in *Adar^Δ7-9^; Mavs^−/−^* animals and the fraction of inflammatory monocytes (CD11b^+^Ly6G^−^Ly6C^hi^) was increased accordingly within the CD11b^+^ cell population (Figure 4C). Intriguingly, normalization to total cell numbers revealed a profound loss of neutrophils and inflammatory monocytes (Figure 4D). Overall, the analysis of these two hematopoietic organs indicates that maintenance of proper B cell and myeloid cell numbers is specifically regulated by ADAR1, independent of inflammation. Interestingly, mice expressing the editing-incompetent *Adar^E861A/E861A^* variant rescued by concomitant deletion of MDA5 show unaltered hematopoiesis (27), suggesting editing-independent functions of the full-length ADAR1 protein in hematopoiesis.

### *Adar^Δ7-9^; Mavs^−/−^* show increased apoptosis of B cells and neutrophils

**Figure 5.**
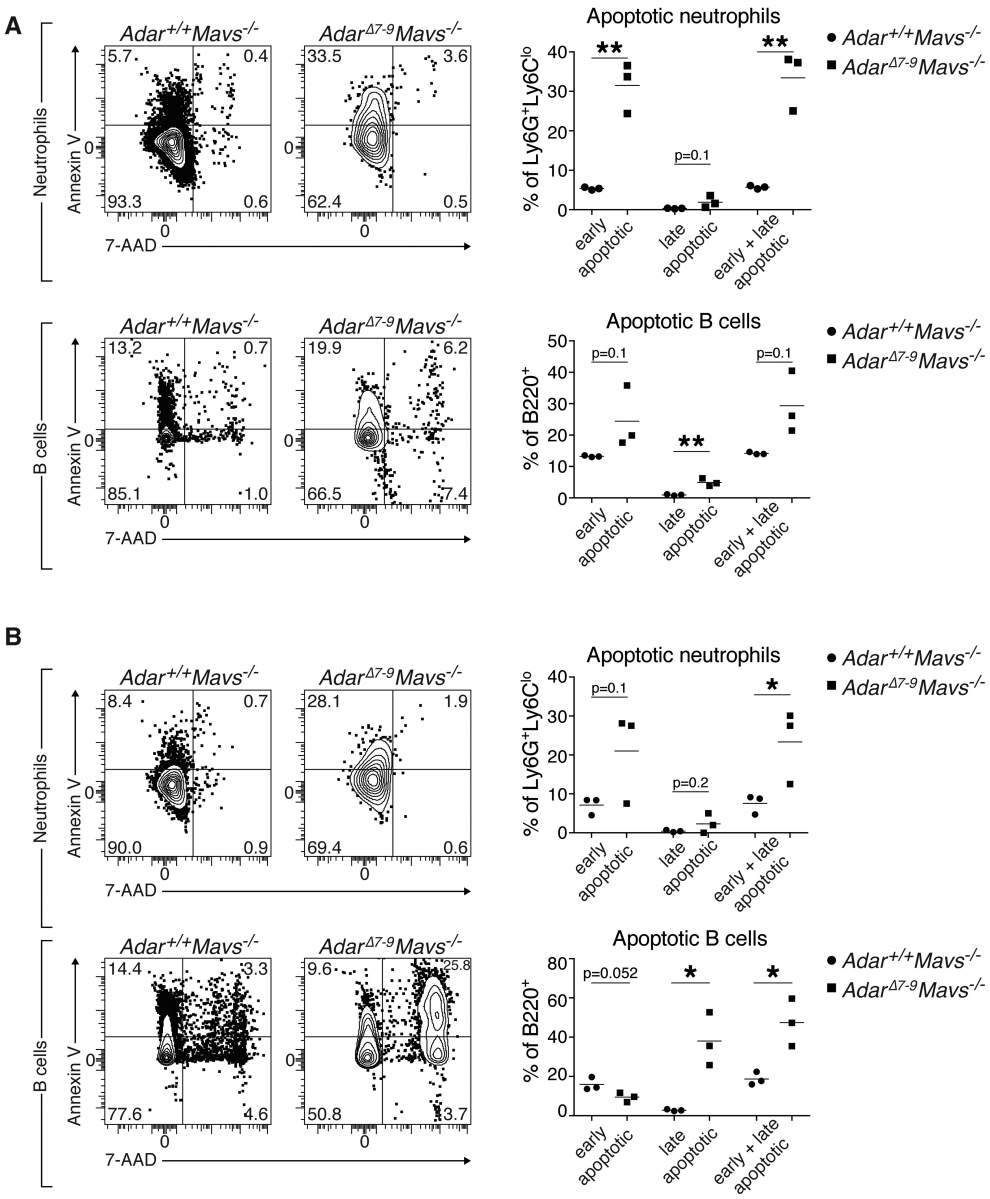
Increased apoptosis in B cells and neutrophils in P15 *Adar^Δ7-9^; Mavs^−/−^* mice. The rate of apoptotic B cells and neutrophils was determined by flow cytometric analysis of Annexin-V and 7-AAD stained cells. (**A**) Bone marrow cells were stained and subgated for neutrophils (CD11b^+^Ly6G^+^Ly6C^lo^, upper panels) and B cells (CD19^+^B220^+^). Left panels show representative flow plots. Numbers within the quadrants indicate percentage within the population. Right dot plots show percentages of early apoptotic (Annexin-V^+^7-AAD^−^), late apoptotic (Annexin-V^+^7-AAD^+^) and the sum of both. (**B**) Analysis of splenocytes performed as described in (A). *n* = 3 mice/genotype; horizontal bars in dot plots represent the mean; *P < 0.05 and **P < 0.01 determined by unpaired Student's *t* test.

Development of B cells was shown to be dependent on ADAR1 in a conditional mouse ablating the ADAR1 protein in these cells. ADAR1 ablation in pre-B cells induces interferon production and apoptosis upon maturation (52). Neutrophil development is strongly dependent on apoptotic mechanisms to control the massive daily generation of these cells (53). The strong reduction of absolute cell numbers of B cells and neutrophils prompted us to analyze apoptotic cell death in bone marrow and spleen of *Adar^Δ7-9^; Mavs^−/−^* animals. Annexin-V staining revealed higher rates of early and late apoptotic neutrophils (subgated for CD11b^+^Ly6G^+^Ly6C^lo^) and B cells (subgated for B220^+^CD19^+^) in the bone marrow (Figure 5A) and the spleen (Figure 5B) of *Adar^Δ7-9^; Mavs^−/−^* animals compared to their littermate controls.

The higher rate of apoptosis in these two cell populations in *Adar^Δ7-9^; Mavs^−/−^* compared to wildtype mice explains the reduction in cellularity of the bone marrow and the spleen. Thus far, initiation of apoptosis in the context of ADAR1 loss was explained by excessive production of interferons, which is rescued upon concomitant deletion of MDA5 (26). The *Adar^Δ7-9^; Mavs^−/−^* animals show only slight upregulation of interferon production, therefore, we conclude that apoptosis is induced by another pathway. Additionally, to the RLR pathway activation, dsRNAs can lead to autophosphorylation of RNA-dependent protein kinase (PKR) and consequently phosphorylation of eukaryotic initiation factor 2α (eIF-2α) without activation of IRF3. This PKR mediated dsRNA recognition was shown to induce global translation attenuation and apoptosis (43,54). During IFN response, ADAR1 also blocks translation attenuation by preventing hyperactivation of PKR (54).

### *Rps3a1* and the pseudogene *Rps3a3* are dysregulated in *Adar^Δ7-9^; Mavs^−/−^* mice

**Figure 6.**
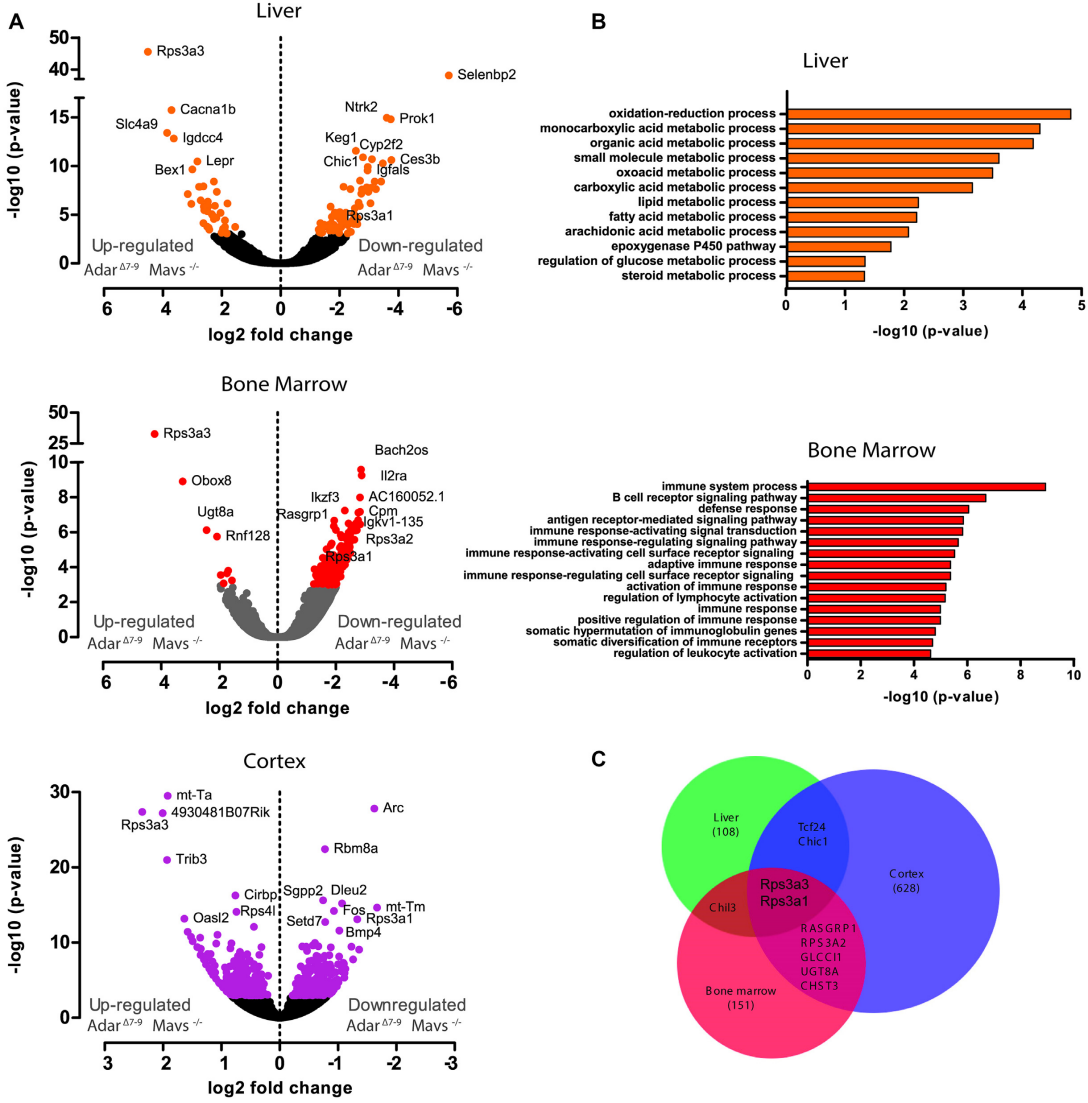
Differentially expressed genes of *Adar^Δ7-9^; Mavs^−/−^* liver, bone marrow and cortex. (**A**) Volcano plots showing differentially expressed genes in liver, bone marrow and cortex of P15 mice. Genes with significantly altered representation(p-value < 0.001) are marked in color. (**B**) Go-terms enriched for significant differentially expressed genes for liver and bone-marrow. (**C**) Common differentially expressed genes of liver, bone-marrow and cortex.

Since the histological analysis of P15 *Adar^Δ7-9^; Mavs^−/−^* showed variable defects in different tissues like the small intestine, colon, spleen or bone marrow (Figure 3D, 4A and B) we wished to identify tissue-specific gene-expression changes underlying these defects. We performed RNA-Seq experiments on bone marrow and liver from P15 *Adar^7-9^; Mavs^−/−^* and used *Adar^+/+^; Mavs^−/−^* littermates as controls. In the liver of *Adar^Δ7-9^; Mavs^−/−^* individuals, a total of 109 genes showed significant differential expression, out of which 66 genes were down-regulated. The down-regulated genes were enriched in liver-intrinsic pathways like oxidation-reduction processes, lipid and fatty acid metabolism and the oxygenase P450 pathway (Figure 6A and B, supplementary data set). Genes up-regulated in the liver of *Adar^Δ7-9^; Mavs^−/−^* mice showed no enrichment for any particular pathway. Differentially expressed genes in the liver also contained genes involved in regulating body size and growth. For instance, insulin growth factor acid labile subunit (*Igfals)* is down-regulated in liver and reported to be involved in regulating body size and growth. *Igfals* knockout mice, show a ∼20% reduction in body size and growth beyond 20 days of birth (55). Interestingly, *Lepr* (leptin receptor) is overexpressed in the liver of *Adar^Δ7-9^; Mavs^−/−^* mice. This gene also regulates body weight and pituitary functions. Mutations in *Lepr* are associated with an obesity phenotype in mice and show elevated plasma levels of leptin, glucose, insulin and corticosterone (56). Another study reported a specific neuronal *Lepr* knockout causes morbid obesity (57). Additionally, we found neurotrophic receptor kinase 2 (*Ntrk2)*, overexpressed in *Adar^Δ7-9^; Mavs^−/−^* mice. This gene is linked to hepatic hematopoiesis and innervation of the liver (58). Out of 109 differentially expressed genes in the liver, 33 (16 upregulated and 17 downregulated in *Adar^Δ7-9^; Mavs^−/−^*) were represented in the interferome database (59). However, these upregulated ISGs showed a log_2_-fold change < 4, indicating a residual, but only mild immune response.

In bone marrow, a dramatic down-regulation of genes was observed. Out of 151 differentially expressed genes only nine were up-regulated in the bone marrow of *Adar^Δ7-9^; Mavs^−/−^* mice (Figure 6A and B, supplementary data set). The down-regulated genes were enriched for immune pathways, B-cell signaling, leukocyte activation and somatic hypermutation of immunoglobulin genes pathways (Figure 6A and B, supplementary data set). While the downregulation of immune pathways may seem in contrast to the observed upregulation of the same pathway in the liver, the downregulation is most likely caused by the above described reduction of neutrophils and monocytes in the bone marrow of *Adar^Δ7-9^; Mavs^−/−^* mice (Figure 4A–D). RasGRP1 was found to be down-regulated in bone-marrow which regulates the development of B1a cells carrying auto-antigen receptors (60). This gene was also down-regulated in the cortex of these mice. We also analyzed RNA-Seq data from the cortex of *Adar^Δ7-9^; Mavs^−/−^* mice (Kapoor, Licht, Jantsch, unpublished). Here we identified a larger number of 628 differentially expressed genes. This high number can most likely be explained by the larger sequencing depth. Here, 306 genes were up-regulated while 322 genes were down-regulated (Figure 6A and B, supplementary data set). Both differentially up- and downregulated genes were enriched for processes that are specific to this tissue, like axon- and neuron development (Supplementary Figure S8). Thus, rescued *Adar^Δ7-9^* mice show tissue-specific changes in gene-expression that correlate with tissue-specific defects observed via histological and cytometry analysis.

Interestingly, two genes were commonly deregulated in all the three tissues examined: RPS3a1, a protein-coding gene was down-regulated while its pseudogene RPS3a3 was found upregulated (Figure 6C). *Rps3a1* encodes a ribosomal protein that is a component of the 40S subunit and has three processed pseudogenes dispersed in the mouse genome, *Rps3a3* is one of them. *Rps3a3* was the most significantly upregulated gene in bone marrow and liver of *Adar^Δ7-9^; Mavs^−/−^* mice while it was the third most significantly upregulated gene in cortex (Figure 6A). Since this ribosomal protein was dysregulated in all examined tissues, we wondered whether translation was altered in *Adar^Δ7-9^; Mavs^−/−^* mice.

### Ribosomal 40S:60S ratios are distorted in *Adar^Δ7-9^; Mavs^−/−^* mice

**Figure 7.**
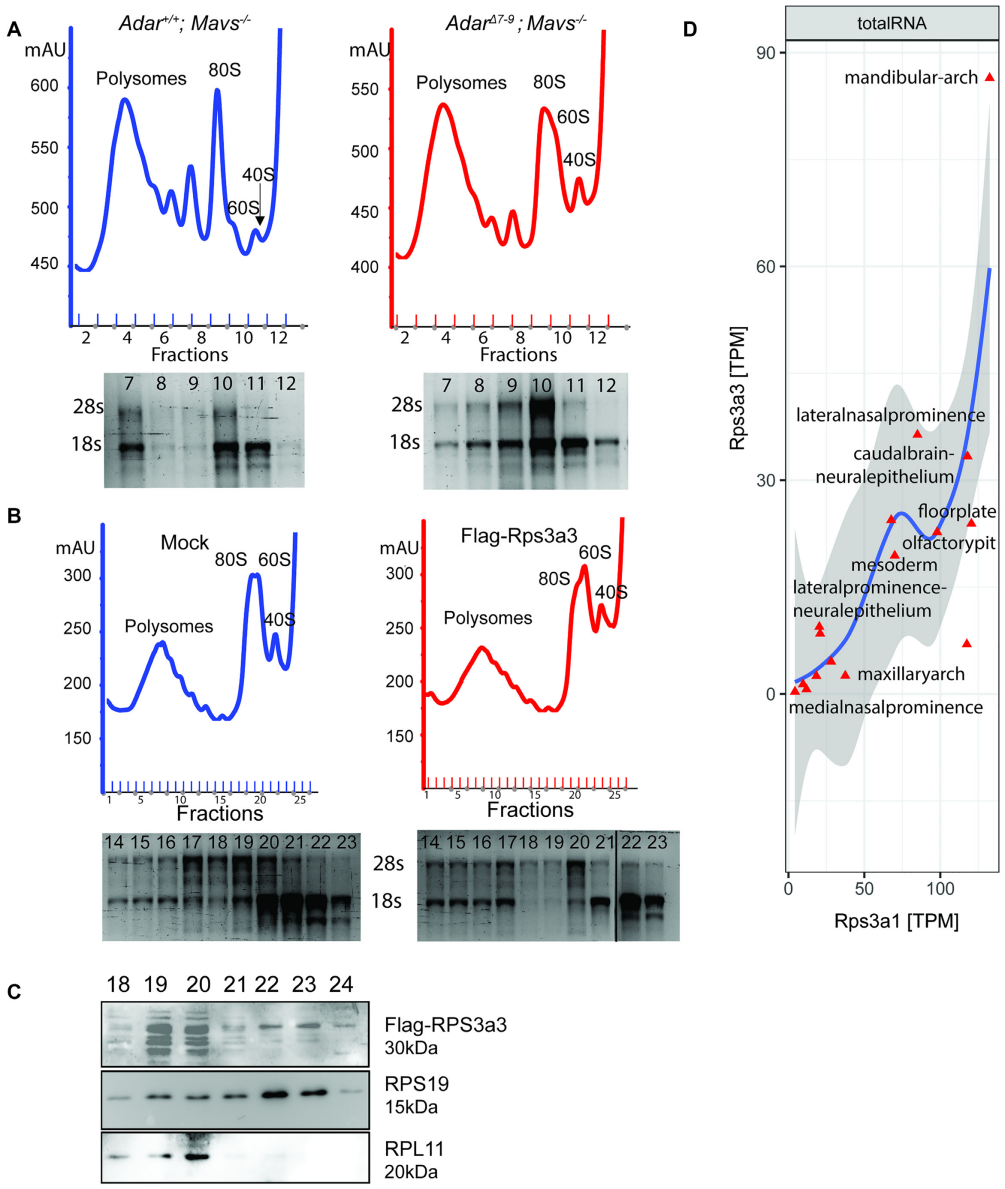
*Adar^Δ7-9^; Mavs^−/−^* show altered ratios of 40S and 60S ribosomal sub-units. (**A**) Polysome profiling of P15 *Adar^Δ7-9^; Mavs^−/−^* and *Mavs^−/−^* liver (upper). RNA from fraction 7–12 loaded on a gel was stained with EtBr to confirm accumulation of 60S particles (lower). (**B**) Polysome profiling of HEK293T cells over-expressing Flag-Rps3a3 compared to mock transfected cells (upper). RNA from fraction 14–23 loaded on EtBr stained gel to confirm ribosomal peaks (lower). (**C**) Western blot of selected fractions from (B) showing incorporation of Flag-Rps3a3 in ribosomal subunits. (**D**) Expression of the pseudogene, *Rps3a3* in wildtype mice in regions of craniofacial tissue at E8.5, E9.5 and E10.5 Data from (41).

To determine the translation profile in *Adar^+/+^; Mavs^−/−^* and *Adar^Δ7-9^; Mavs^−/−^* mice, we performed polysome profiling on cell lysates of livers isolated from P15 mice. The polysome profile of *Adar^Δ7-9^; Mavs^−/−^* livers showed a decrease in 80S monosomes at the expense of an increase of 60S and to some extend 40S particles (Figure 7A). This is consistent with a previous report showing that knock-down of *Rps3a1* in HeLa cells causes accumulation of free 60S particles resulting from a strong alteration in 40S subunit production due to a failure in the processing of 18S rRNA (61).

*Rps3a3* is an intron-less processed pseudogene, likely derived from the parental gene *Rps3a1*. *Rps3a3*, shows nine mismatches in its nucleotide sequence compared to the coding region of *Rps3a1* which would result in two amino acid changes at the protein level (Supplementary Figure S7A). Also, *Rps3a3* has an annotated promoter. This led us to investigate whether *Rps3a3* could form a protein product. To achieve this, we cloned *Rps3a3* with an N-terminal FLAG-tag and C-terminally fused to eGFP with a self-cleaving 2A peptide. Transfection of this construct in HEK293T, showed the presence of GFP indicating that Flag-RPS3a3 was translated (Supplementary Figure S7B). Expression of full-length Flag-RPS3a3 was confirmed by western blotting (Supplementary Figure S7B). Since the putative protein product of RPS3a3 is similar to RPS3A1 differing in only two amino acids, we checked the effect of over-expression of Flag-RPS3a3 by polysome profiling. We overexpressed Flag-RPS3a3 in HEK293T since RPS3a3 is not conserved in humans. Over-expression of RPS3a3 resulted in an accumulation of free 40S and 60S particles and a decrease in 80S particles (Figure 7B). Western blot analysis on the fractions of polysome profiling confirmed Flag-RPS3A3, to be present in the ribosomal fractions (Figure 7C). Fractions corresponding to 60S and 40S were verified by probing for *Rps19* (a SSU protein) and Rpl11(a LSU protein). Mock transfected cells did not show any bands after probing with Flag antibody (Supplementary Figure S9). The effect of over-expression of RPS3a3 (Figure 7B) and down-regulation of RPS3a1 correlates with the polysome profile of *Adar^Δ7-9^; Mavs^−/−^* mice where we observed both 60S and 40S to be accumulating compared to *Mavs^−/−^* mice. Thus, a de-regulated RPS3a1 and RPS3a3 might affect the ribosomal subunits of *Adar^Δ7-9^; Mavs^−/−^* mice.

We could not detect expression of *Rps3a3* in the tissues of *Mavs^−/−^* littermates. However, RNA-Seq analysis of wild-type mice at stages E8.5, E9.5 and E10.5 showed high expression of the pseudogene *Rps3a3*, in various micro-regions involved in cranio-facial development (41). Highest expression was seen in mandibular arch at these early embryonic stages of wild-type mice, with levels as high as 90 transcripts per million (TPM) (Figure 7D). Expression of *Rps3a3* and *Rps3a1* was highly correlated in these tissues (Figure 7D). Analysis of the ENCODE RNA-Seq data from wildtype mice at 10 weeks of age and at P0 indicated no expression of *Rps3a3* with TPM close to zero. All these results confirm that *Rps3a3* is not expressed at post-natal stages in wildtype mice in the tissues studied and is only expressed in the micro-regions of facial structures at embryonic stages E8.5, E9.5 and E10.5. While in *Adar^Δ7-9^; Mavs^−/−^* pups, expression of the pseudogene is de-regulated and shows a high expression in liver, bone marrow and cortex.

### Dysregulation of *Rps3a1* and *Rps3a3* is not dependent on editing

**Figure 8.**
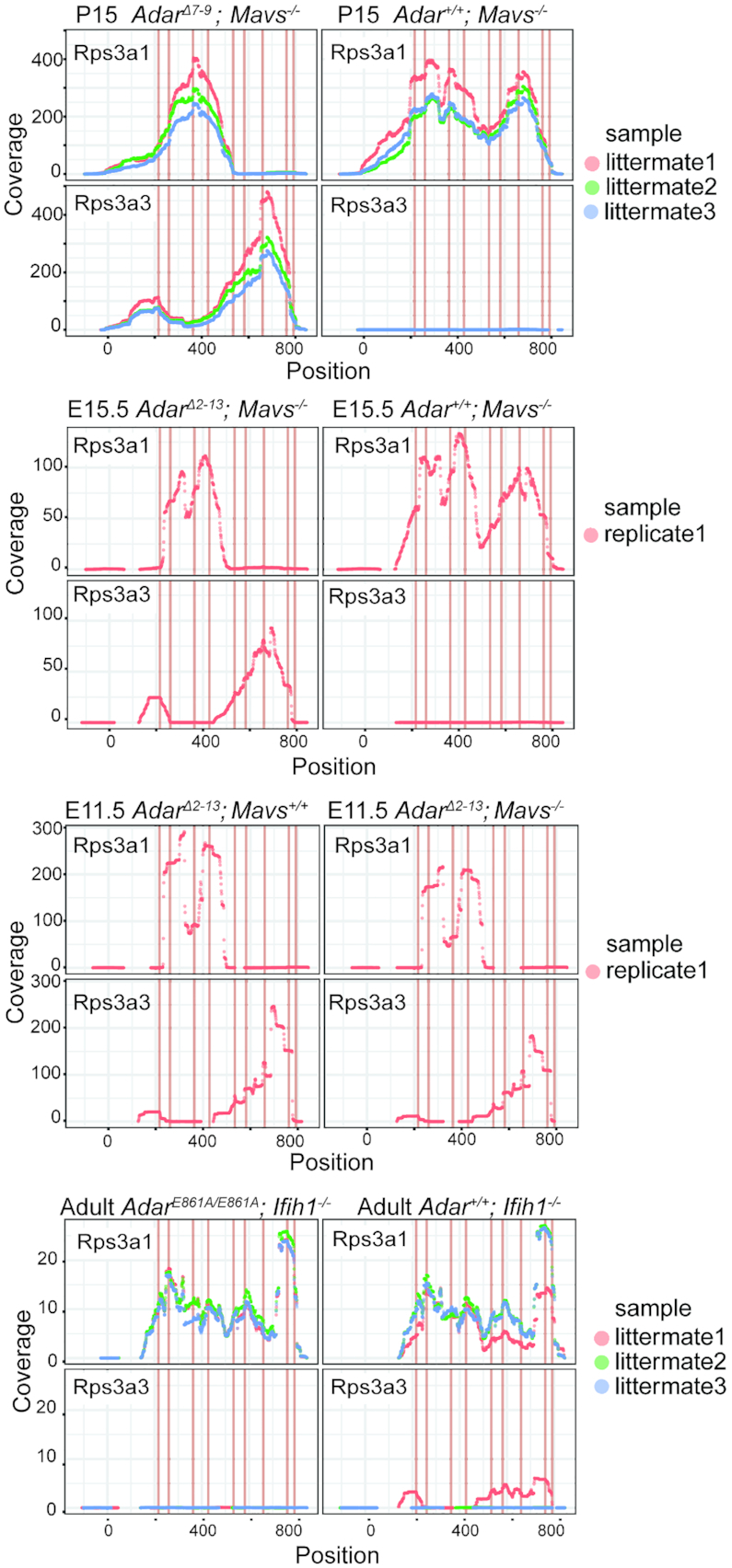
Read coverage over *Rps3a1* and *Rps3a3* in rescued *Adar^Δ7-9^, Adar^Δ2-13^* and *Adar^E861A/E861A^* RNA-Seq read coverage over the UTR and coding regions of *Rps3a1* and *Rps3a3* in livers of P15 *Adar^Δ7-9^; Mavs^−/−^* and *Mavs^−/−^*, in E15.5 *Adar1^Δ2-13^; Mavs^−/−^*, E15.5 *Adar1^+/+^; Mavs^−/−^*, E11.5 *Adar1^Δ2-13^; Mavs^+/+^*, adult *Adar1^E861A/E861A^; Ifih1^−/−^* and adult *Adar1^+/+^; Ifih1^−/−^*. Red lines in the graphs indicate the mismatches between *Rps3a1* and *Rps3a3*.

*Rps3a1* is located on chromosome 3 within 10MB of *Adar*. We therefore examined if the genetic variability arising from the 129/SV-derived congenic interval surrounding the *Adar^Δ7-9^* knockout site could account for the different *Rps3a1* expression. Analysis of E11.5 whole-embryo RNA-Seq data of another *Adar^Δ2–13^* and *Adar^Δ2–13^*; *Mavs^−/−^* strain that was maintained in an C57Bl6N background (28), showed that *Rps3a1* and *Rps3a3* are also dysregulated in both these *Adar* allele bearing strains, irrespective of the background (Figure 8). This supports the idea that dysregulation of *Rps3a3* and *Rps3a1* is most likely caused by *Adar-*deficiency and is not dependent on the mouse strains investigated. Interestingly, the two *Rps3a* isoforms are not dysregulated in the brain lysates of 12 week old mice expressing full-length but catalytically dead *Adar^E861A/E861A^*; *Ifih1^−/−^* mice (27). This strongly suggests that dysregulation of *Rps3a1* and *Rps3a3* is not caused by a lack of editing but rather by other activities of ADAR1 such as RNA-binding (Figure 8). This notion is also supported by the finding that no editing events could be detected in RNAs encoding *Rps3a1* or *Rps3a3* in cortex, liver or bone-marrow of P15 pups {Licht, 2019 #8438}.

## DISCUSSION

All existing deletion- and point mutation alleles of *Adar* show a comparable embryonic lethal phenotype that is accompanied by liver disintegration and heightened immune response. However, as the different alleles of *Adar* produce various fragments of the protein, they may also affect so far unstudied functions of *Adar*. Indeed, attempts to rescue the various *Adar* alleles by either deleting *Mavs* or *Ifih1* leads to different phenotypes, depending on the *Adar* allele used (24,27–29). Since, the point mutation *Adar^E861A/E861A^; Ifih1^−/−^* shows a complete rescue of lethality and the complete deletion *Adar^Δ2-13^; Mavs^−/−^* survives only 24 hours post-partum, it appears that editing-independent functions of ADAR1 are important for normal growth and development in mice. This notion is also consistent with the finding that deletion of the interferon-inducible long variant of *Adar p150* cannot be rescued by expression of the constitutively expressed version *Adar p110* (24). Known editing-independent functions of ADAR1 most likely result from the RNA-binding activity of ADARs that may regulate RNA stability, microRNA biogenesis, splicing and translation (5,7,8,20,21). However, the molecular mechanisms causing post-natal lethality and miniature size of *Adar^Δ2-13^; Mavs^−/−^* mice remain unknown.

The two deletion alleles *Adar^Δ2-13^* and *Adar^Δ7-9^* were always considered functionally identical and have indeed been used interchangeably. Both alleles show embryonic lethality at E12.5. However, our study shows that *Adar^Δ7-9^* pups rescued by deletion of *Mavs* live significantly longer than *Adar^Δ2-13^; Mavs^−/−^* pups. This discrepancy can most likely be explained by the fact that the *Adar^Δ7-9^* allele can form a truncated, editing-deficient protein. Interestingly, deletion of exons 7–9 in mouse Adar removes the region where the nuclear localization signal is located in human *Adar*. Our study shows that a mouse protein corresponding to truncated p110*^Δ7-9^* remains primarily nuclear. This suggests that human and mouse *Adar* may differ in their NLSs. In fact, a critical amino acid for NLS function (R801) is unique to the human but not the mouse protein (48). As expected, p150*^Δ7-9^* shows cytoplasmic localization.

Interestingly, the *Adar^Δ7-9^* allele had been analyzed previously (29). In this case, a different *Mavs* allele was used for the rescue of the *Adar^Δ7-9^* allele. In the mentioned study, deletion of *Mavs* only resulted in a 1 to 2 day survival *post partum*. In contrast, the mice analyzed by us survive significantly longer. At present, we cannot address whether this is a result of the different Mavs alleles or a result of different strain backgrounds. The mice used in this study had a ∼75% Sv129 and 25% Bl6 background. It is therefore possible that the different backgrounds used contribute to the observed different phenotypes. We can almost certainly exclude differences due to animal housing conditions as mice analyzed in this study were kept in different mouse houses in different facilities over several years and showed comparable litter sizes and survival rates. Moreover, we find sporadic long-term survivors in our litters. These long-term survivors catch up in growth and look normally, at least by histological examination. Unfortunately, the long-term survivors occur at very low frequency, precluding their systematic investigation. In any case, the occurrence of long-term survivors indicates that the penetrance of the *Adar^Δ7-9^* phenotype in a *Mavs* background may be variable.

The presence of the residual *Adar^Δ7-9^* protein may have different effects. Possibly the remaining dsRBDs of *Adar^Δ7-9^* may shield dsRNAs thereby preventing them from excessive activation of MAVS. A similar function has been postulated for the catalytic dead version, ADAR^E861A^. This mutant can be fully rescued by a deletion of *Ifih1* indicating that RNA-binding of ADAR1 may have critical functions (26). Also the Z-DNA binding domains may interfere with other RNA-processing steps such as PKR activation, miRNA processing, or transposon activation, to name a few. All these functions have been previously reported for ADARs as editing-independent functions (9,10,62,63). In fact, PKR has been shown to be critically involved in the sensing of dsRNAs, particularly in the absence of ADARs (62,64,65). Consequently, it will be interesting to determine whether a concomitant deletion of PKR may further alleviate the phenotype of the *Adar^Δ7-9^; Mavs^−/−^* mice.

The phenotype of *Adar^Δ7-9^; Mavs^−/−^* mice may also be caused by defects in the production of ribosomal subunits due to dysregulation of *Rps3a1* and *Rps3a3*. This, in turn, may affect translation of mRNAs. As both, *Adar^Δ2–13^; Mavs^−/−^* and *Adar^Δ7-9^; Mavs^−/−^* mice show a deregulated expression of *Rps3a1* and its pseudogene *Rps3a3* in all tissues examined at embryonic as well as at post-natal stages, it may be a possible cause for the early lethality observed in both mouse strains. At present, we cannot judge whether the different extents of rescue observed between the two deletion alleles is also due to different expression levels of *Rps3a1* and its pseudogenes. Still, to our knowledge this is the first report that demonstrates that ADAR can affect ribosomal subunit levels.

The fact that *Rps3a1* and *Rps3a3* de-regulation was observed in both *Adar^Δ7-9^* and *Adar^Δ2-13^* mice but not in the *Adar^E861A/E861A^* allele, argues for an editing-independent regulation of these ribosomal genes. We cannot exclude the possibility that upregulation of *Rps3a3* is the result of down-regulated *Rps3a1*, or vice-versa. Most interestingly, we could also show that the annotated pseudogene *Rps3a3* has indeed the potential to form a protein product that can get incorporated in ribosomal subunits (Figure 7C). Whether this would also be the situation in vivo remains open. We have also tested whether editing of miRNAs might affect *Rps3a1* expression. However, so far, no miRNAs that target the mRNAs encoding this small ribosomal subunit protein have been identified (66).

RPS3a1 has extra-ribosomal functions and has been shown to be involved in cell cycle regulation, cell apoptosis, immunity and signal transduction (67–69). Enhanced *Rps3a1* expression primes cells for apoptosis and suppression of such enhanced expression leads to execution of apoptosis (69). Knock-down of *Rps3a1* triggers death of undifferentiated HL-60 cells but not of retinoid-induced differentiated cells. This is consistent with our observation, *Adar^Δ7-9^; Mavs^−/−^* show drastically reduced mature B-cells along with massive apoptosis while the numbers of immature B-cells do not change compared to *Mavs^−/−^* mice. Most interestingly, *Drosophila* and zebra fish mutants with knock-down of *Rps3a1* show defects in body size and hematopoiesis as seen in *Adar^Δ7-9^; Mavs^−/−^* mice (70–72). Given these results, it is interesting to speculate if defects in apoptosis, hematopoiesis and body size observed in rescued *Adar^Δ7-9^* and *Adar^Δ2-13^* are due to down regulated Rps3a1.

## DECLARATION ETHICS STATEMENT

All experiments were approved by the institutional ethics committee and performed in accordance with the Austrian law for animal experiments (BGBl. I Nr. 114/2012), and in accordance to the guidelines recommended by the German Society of Laboratory Animals (GV-SOLAS). Animal experimental protocols were approved and authorized through the permission BMWFW-66.009/0267-WF/V/3b/2017 issued by the Austrian Ministry of Science.

## DATA AVAILABILITY

RNA-Seq data generated in this study has been deposited in the European Nucleotide Artiche under project numbers PRJEB31568 and PRJEB31565.

## Supplementary Material

gkaa025_Supplemental_Files
