# Supplementary material for: An internal deletion of ADAR rescued by MAVS deficiency leads to a minute phenotype
Source: Nucleic Acids Res. 2020 Jan 20;48(6):3286–303. doi: 10.1093/nar/gkaa025 (PMC7102943; doi:10.1093/nar/gkaa025)
Supplement: gkaa025_Supplemental_Files [file gkaa025_supplemental_files.zip › Supplements28_12_2019.pdf]

A

Parents: *Adar*$^{+/\Delta 7\text{-}9}$; *Irf3*$^{-/-}$						
			Observed			Expected
Day	Litters	Embryos	*Adar*$^{+/+}$	*Adar*$^{+/\Delta 7\text{-}9}$	*Adar*$^{\Delta 7\text{-}9}$	*Adar*$^{\Delta 7\text{-}9}$
E11.5	1	9	1	6	2	2.25
E13.5	2	18	4	11	3	4.5
E14.5	2	14	2	10	2	3.5
Born	5	21	6	15	0	5.25

B

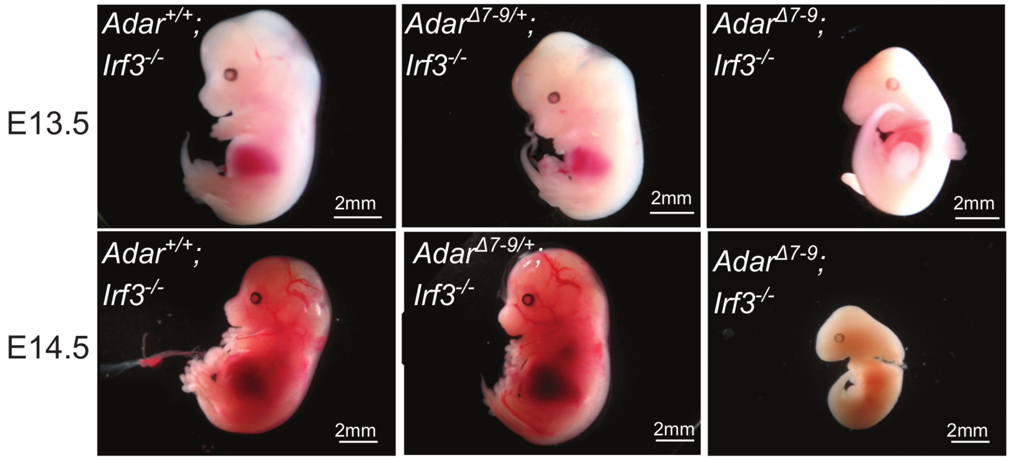

C

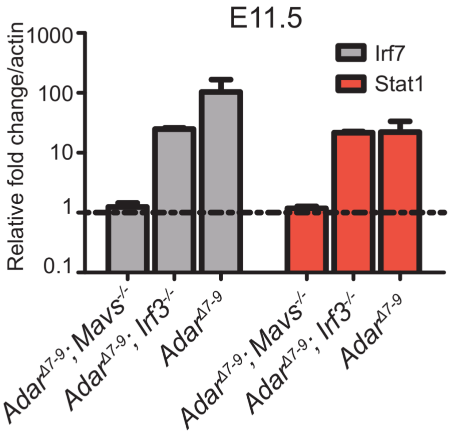

Figure S1: **A**) Survival of Adar / Irf3 knockout mice at different stages of development obtained by crossing Adar $^{+/\Delta 7\text{-}9}$; Irf3$^{-/-}$ mice. **B**) Picture of embryos at E13.5 and E14.5, obtained by crossing Adar $^{+/\Delta 7\text{-}9}$; Irf3. At E13.5 and E14.5 Adar $^{\Delta 7\text{-}9}$; Irf3$^{-/-}$ mice are developmentally retarded.
C) Expression of Stat1in Adar $^{\Delta 7\text{-}9}$, Adar $^{\Delta 7\text{-}9}$; Mavs $^{-/-}$ and Adar $^{\Delta 7\text{-}9}$; Irf3$^{-/-}$ at E11.5. The horizontal black dotted line indicates the immune response in corresponding littermates, wild-type for Adar .

A

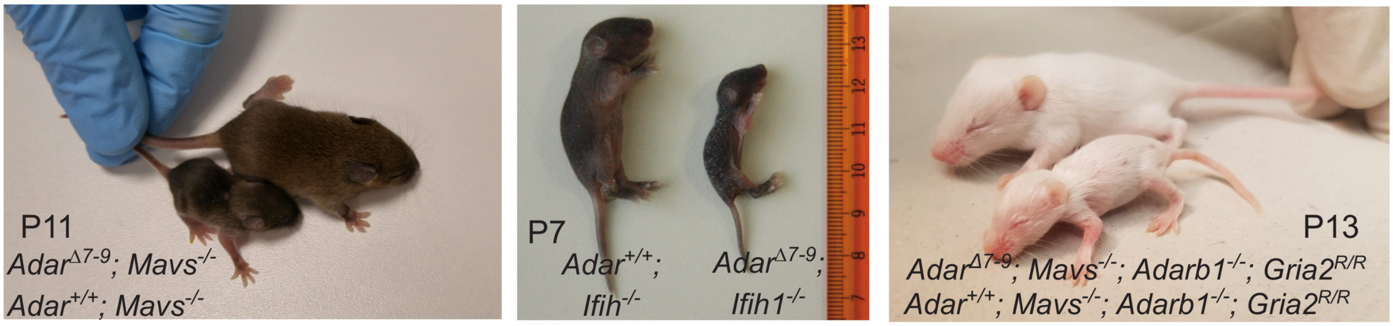

B

			Observed			Expected
Genotype	Litters	Pups	*Adar*$^{+/+}$	*Adar*$^{+/\Delta 7\text{-}9}$	*Adar*$^{\Delta 7\text{-}9}$	*Adar*$^{\Delta 7\text{-}9}$
Mavs$^{-/-}$	13	105	35	57	13	26.25
Ifih1$^{-/-}$	15	134	45	77	12	33.5
Adarb1$^{-/-}$ ***Mavs***$^{-/-}$	13	76	22	37	17	19

Figure S2: A) Images of *Adar* $^{\Delta 7\text{-}9}$; *Mavs*$^{-/-}$, *Adar*$^{\Delta 7\text{-}9}$;*Ifih1*$^{-/-}$ and *Adar*$^{\Delta 7\text{-}9}$;*Mavs*$^{-/-}$; *Adarb1*$^{-/-}$;*Gria2*$^{R/R}$ with their corresponding wildtype littermates. **B)** Genotype ratios of the progeny obtained by crossing *Adar*$^{+/\Delta 7\text{-}9}$ into different genetic backgrounds.

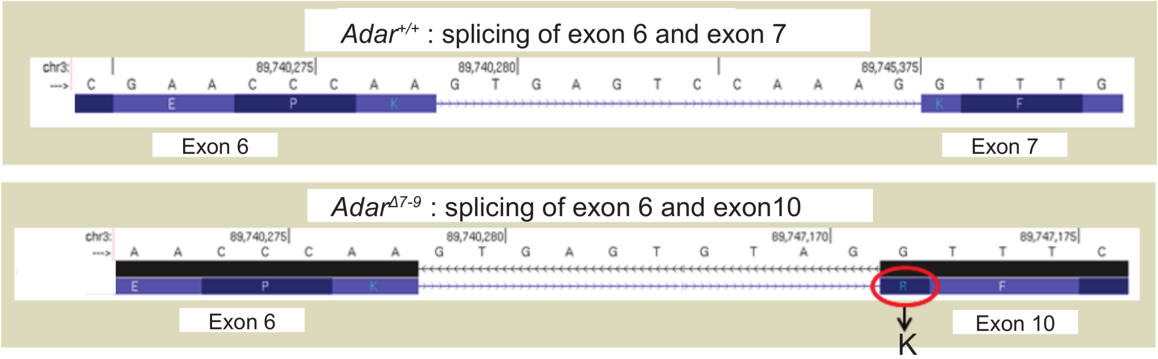

Figure S3: Modified UCSC browser image showing that splicing between exon 6 and exon 7 in *Adar* generates an AAG coding in the wild-type. In the *Adar*$^{\Delta 7\text{-}9}$ allele exons 6 and 10 can splice and have the potential to generate in in-frame AAG codon and thus a trunctated protein (introns were truncated for better depiction). The black box in the Adar$^{\Delta 7\text{-}9}$ sequeunce has been verified by Sanger sequencing of the RT-PCR product shown in Figure 2B.

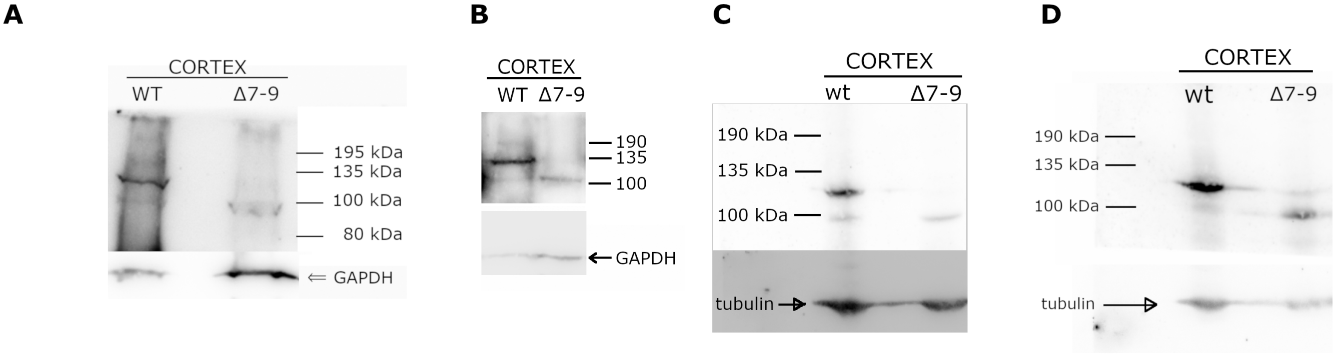

Figure S4: Detection of full-length and Δ7-9 ADAR1 p110 in cortex tissue lysates of 4 independent mice. Lysates of different mouse cortices were generated (left, right), separated on 8% SDS polyacrylamide gels, blotted, and detected with an antibody against ADAR1 (top) and GAPDH (A, B; bottom) or tubulin (C,D; bottom).

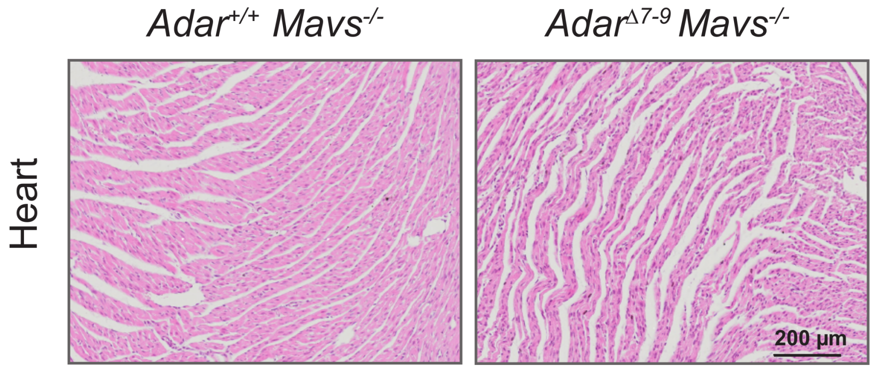

Figure S5: Histological section of hearts isolated from *Adar*$^{\Delta 7\text{-}9}$; *Mavs*$^{-/-}$ (left) and *Adar*$^{+/+}$; *Mavs*$^{-/-}$ (right) mice at P15 and stained with H&E.

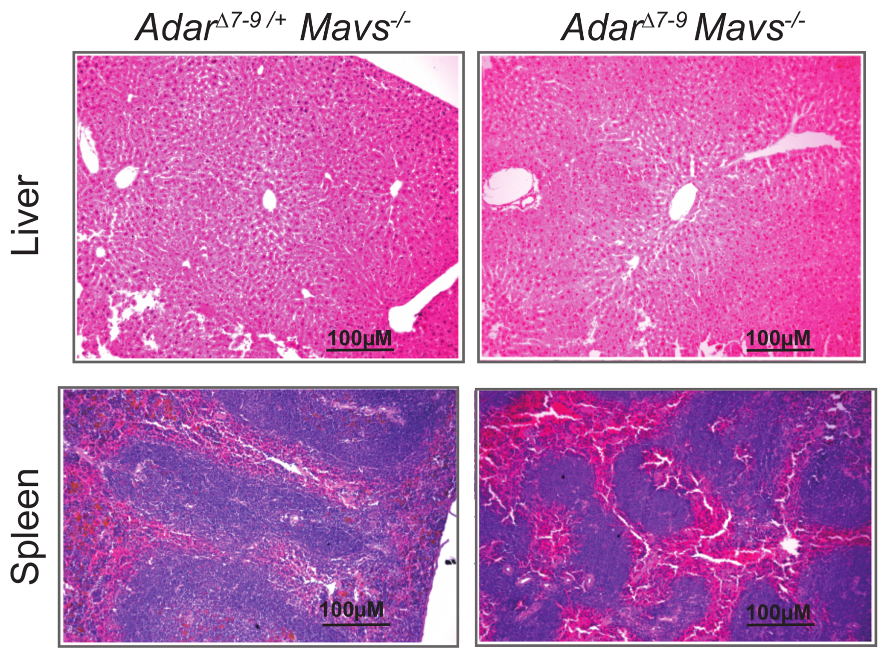

Figure S6: Histological sections of the liver and spleen of 18 months old *Adar*$^{\Delta7\text{-}9}$; *Mavs*$^{-/-}$ and *Adar*$^{+/+}$; *Mavs*$^{-/-}$ mice stained with H&E.

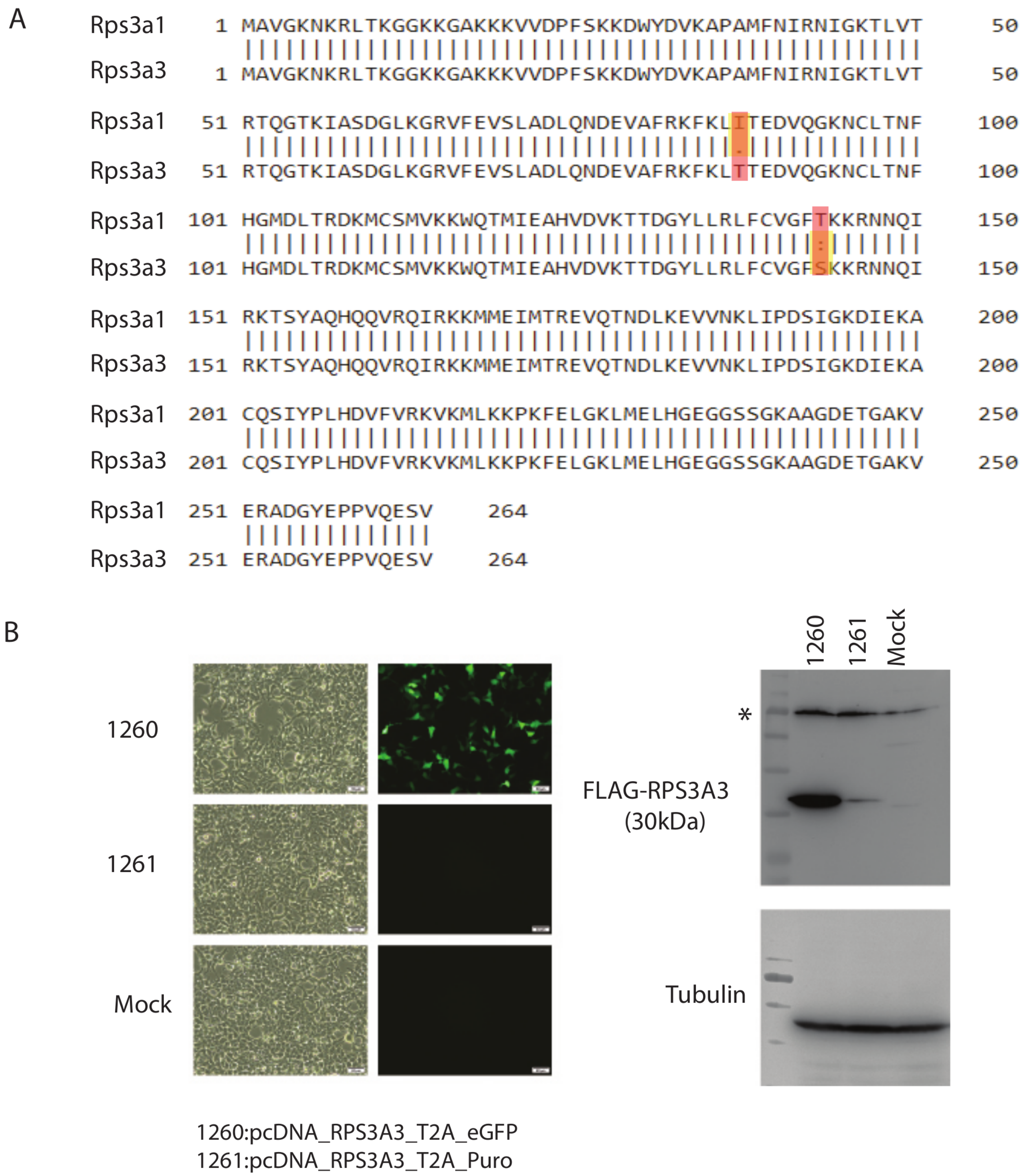

Figure S7: **A)** Alignment of putative translation products of RPS3A1 and RPS3A3. Mismatches between the two proteins are highlighted. **B)** A cDNA encoding RPS3A3 was cloned either as a Flag-tagged version fused to self-cleaving GFP, (clone1260), or to a puromycin ORF, (clone 1261). Upon transfection into HEK293 cells the presence of the eGFP protein was visualized (left). Western blot with an anti-FLAG antibody demonstrates expression of the RPS3A3 fudion in clone 1260, while almost no protein was detected in clone 1261. Asterisk denotes unspecific background band.

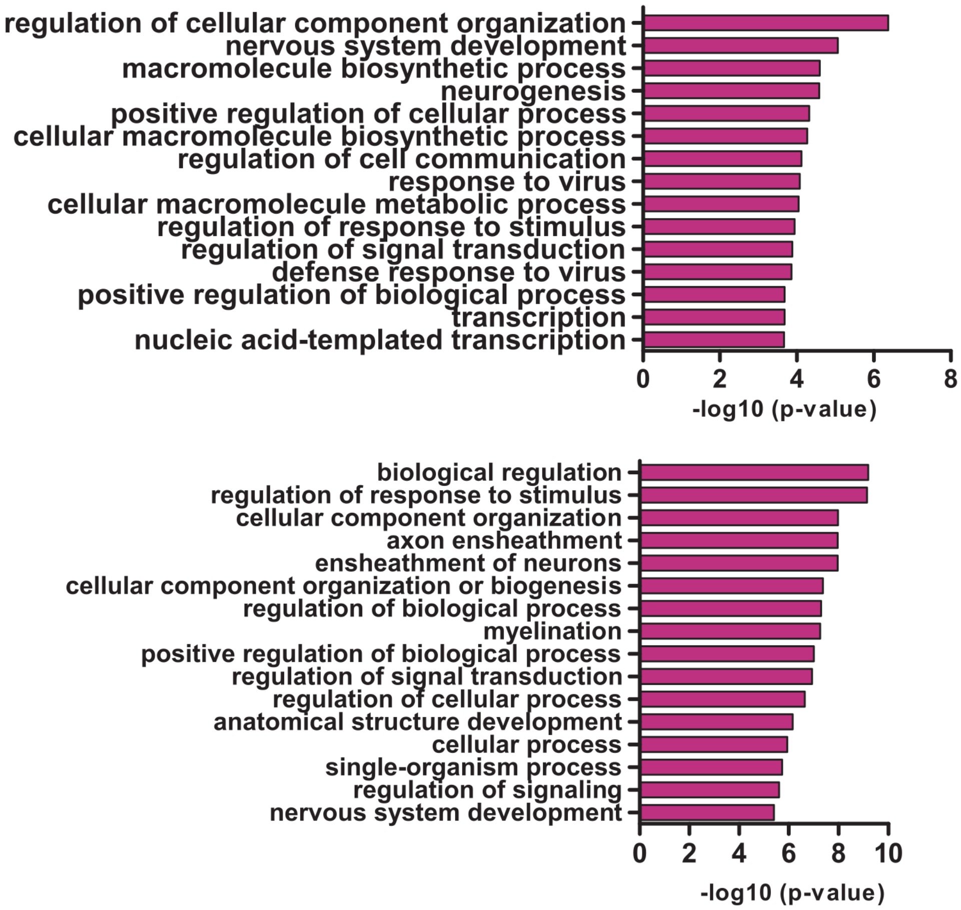

Figure S8: Bar graphs showing GO terms enriched in mRNAs expressed in the cortex of p15 ADARΔ7-9; MAVS-7- mice.
Top panel: Significantly down regulated genes (top 20%).
Bottom panel: Significantly up-regulatd genes (top 15%).

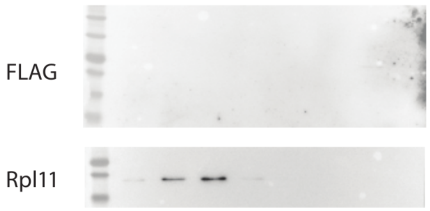

Figure S9: Western blot of fractions of polysome profiles of mock-transfected cells. Fractions were tested for the presence of RPL11 (bottom) and FLAG-tagged protein (top).

chromosome	position	gene name	original nt	alt nt	detected in wt	detected in Δ7-9	repeat	editing rate wt	editing rate Δ7-9
chr2	5019723	Optn	T	C	yes	no	no	0,405	0,000
chr4	155621903	Slc35e2, Gm16023, Gm10563, Cdk11b	A	G	yes	no	yes	0,439	0,000
chr12	54672357	Eapp	T	C	yes	no	yes	0,444	0,000
chr16	32300288	Rnf168	A	G	yes	no	yes	0,467	0,000
chr5	87124829	Ugt2b5	T	C	yes	no	no	0,468	0,000
chr5	87124786	Ugt2b5	T	C	yes	no	no	0,517	0,000
chr4	21834548	Usp45	A	G	yes	no	yes	0,529	0,000
chr14	21796764	Samd8	A	G	yes	no	yes	0,552	0,000
chr5	125457476	Bri3bp	A	G	yes	no	yes	0,572	0,000
chr7	140135727	Paox, Mtg1	A	G	yes	no	no	0,659	0,000

Table S1: The ADAR1 Δ7-9 allele is editing deficient.
A selection of 10 positions in the mouse transcriptome that are abundantly edited in wild-type mice completely lack editing in mice expressing the ADAR1 Δ7-9 allele.
Chromosome indicates the chromosome where the editing site is located. Position marks the position of the edited nucleotide on the respective chromosome. Gene name indicates the name of the gene the edited position resides in. Original and alt nt marks the genomically encoded nucleotide (T or A) and the alternative nucleotide found in the transcript (C or G). Detected in wt marks whether the editing event is detected in wt mice. Detected in Δ7-9 indicates whether the editing event is detected in mutant mice. "Repeat" indicates whether the editing event overlaps with a repetitive sequence. "Editing rate wt and Δ7-9" indicates editing rates in wt and Δ7-9 mice respectively.
Editing sites were selected when the position was covered by >10 reads in 2 out of 3 replicates for wt and Δ7-9 mice.
